# Quantifying the Influence of Nanosheet Aspect Ratio on Network Morphology and Junction Resistance in Solution-Processed Nanosheet Networks

**DOI:** 10.1021/acsnano.5c04008

**Published:** 2025-09-11

**Authors:** Eoin Caffrey, Jose M. Munuera, Cian Gabbett, Luke Doolan, Joseph Neilson, Rebekah A. Wells, Mark McCrystall, Alexandra McNamara, Tian Carey, Martin Gerlei, Paul Seifert, Georg S. Duesberg, Jonathan N. Coleman

**Affiliations:** † School of Physics, CRANN & AMBER Research Centres, 8809 Trinity College Dublin, Dublin 2, Ireland; ‡ Instituto de Ciencia y Tecnología del Carbono, NCAR-CSIC, C/Francisco Pintado Fe 26, Oviedo 33011, Spain; § Institute of Physics, University of the Bundeswehr Munich, Werner-Heisenberg-Weg 39, Neubiberg D-85579, Germany

**Keywords:** graphene, aspect ratio, junction resistance, nanosheet network morphology, spray coating

## Abstract

Optimizing solution-processed nanosheet networks for electronic applications requires understanding the relationship between nanosheet dimensions, network morphology, and electrical properties. Here, we fabricate graphene nanosheets with both low- and high-aspect-ratios using liquid-phase exfoliation (LPE) and electrochemical exfoliation (EE), respectively. Spray-coated networks of both nanosheet types display distinct morphological and electrical properties. High-resolution 3D imaging shows that low-aspect-ratio LPE nanosheet networks display a disordered, porous structure with point-like junctions. Conversely, high-aspect-ratio EE graphene forms low-porosity networks with highly aligned nanosheets with large-area conformal junctions. Electrical measurements demonstrate that EE networks achieve lower resistivity and reduced percolation thicknesses due to reduced junction resistances and improved nanosheet alignment. We propose a theoretical model linking nanosheet bending rigidity, aspect ratio, and junction formation, highlighting the critical role of nanosheet flexibility in enabling conformal junctions. Furthermore, by size-selecting both nanosheet types, we measure the dependence of network resistivity on nanosheet thickness. LPE networks show increasing resistivity with thickness, whereas EE networks exhibit decreasing resistivity. We develop a simple model linking these behaviors to point-like and planar junctions respectively and quantify the size-dependence of both nanosheet and junction resistance for both cases. Unexpectedly, data analysis using this model predicts the EE nanosheets to be more conductive than the LPE ones, a fact confirmed by THz spectroscopy. This study establishes the importance of nanosheet aspect ratio and flexibility in governing network morphology and electrical performance. Our findings provide key insights for developing high-performance, solution-processed 2D material networks for future electronic devices.

## Graphical abstract

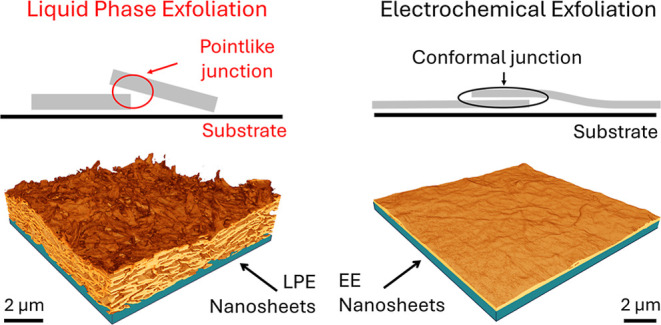

## Introduction

Inks containing 2D nanosheets can be solution-deposited to form thin films which are often referred to as networks.^1,2^ Such nanosheet networks find uses across a wide variety of applications, including energy storage,^2,3^ biosensing^4^ and catalysis.^5^ In particular, solution-processed nanosheet networks have shown promise for use in flexible and wearable electronic applications,^6–8^ demonstrating a range of devices including transistors,^9^ photodetectors^10^ and piezoresistive sensors.^11,12^ These applications typically require the network conductivity or mobility to be as large as possible.^1,13^ Hence, there is significant interest in developing our understanding of how to maximize the electrical properties of these systems.

A key distinction between charge transport through networks of nanosheets versus single nanosheet devices (e.g., produced from mechanical exfoliation) is the presence of internanosheet junctions.^1,14^ These junctions add extra resistive elements to the network, increasing the overall network resistance and reducing conductivity and mobility.^14–16^ The effect of junction resistance can be understood by realizing that, because charge carriers must cross a junction every time they traverse a nanosheet, the total network resistance must be proportional to *R*_NS_ + *R*_J_, where *R*_NS_ and *R*_J_ are the nanosheet and junction resistances, respectively. This concept has been used to establish models for both conductivity and mobility in networks of 2D nanosheets, as well as 1D nanowires/nanotubes and 0D nanodots.^14^ These models clearly show that the network mobility and conductivity can be maximized toward their intrinsic, individual particle values by reducing *R*_J_ as much as possible, ideally to a value below that of *R*_NS_. This means that the ability to engineer junction properties, and hence to control their resistances, is extremely important if one is to maximize the electrical performance of printed networks.

It is becoming clear that the resistance of junctions is connected to the morphology of the network. For example, networks of aligned nanosheets tend to have high conductivity or mobility,^9,13,15,17–19^ while the opposite is true for disordered networks.^20–23^ This may be because networks of highly aligned nanosheets have large-area junctions and so reduced junction resistances. Moreover, highly aligned nanosheet networks seem to form more readily for nanosheets with high aspect ratio, *k*_NS_, which is described by the ratio of nanosheet length, *l*_NS_, and thickness, *t*_NS_, as *k*_NS_ = *l*_NS_/*t*_NS_. Such high aspect ratio nanosheets are often produced in solution by electrochemical exfoliation (EE)^13,14,17,24^ or are produced by exfoliation and reduction of graphene oxide.^18,25,26^ Based on these observations, we believe that quantifying the factors that link nanosheet aspect ratio to network morphology, junction resistance, and electrical conductivity will significantly advance our understanding of these systems and facilitate enhanced device performance.

Here, we study the effect of nanosheet aspect ratio on the morphology and electrical properties of printed nanosheet networks. Graphene nanosheets produced by both liquid-phase exfoliation (LPE) and EE are used as model systems of low-aspect-ratio and high-aspect-ratio nanosheets, respectively. We use spray coating to produce networks of both nanosheet types for a range of network thicknesses, as well as a range of nanosheet sizes. 3D imaging is combined with electrical measurements to fully determine the connection between nanosheet aspect ratio, network morphology and junction resistance. This allows us to quantitatively measure and develop models to describe the factors controlling resistivity in solution-processed nanosheet networks.

## Results and Discussion

### Ink Characterization

Graphene nanosheets were exfoliated using both LPE and EE as described in Methods. The resultant inks contain nanosheets, with a broad range of sizes, suspended in IPA and are sometimes referred to as stock dispersions. Photographs of inks containing EE and LPE graphene nanosheets are shown in Figure 1A. These inks are colloidally stable for ∼2 weeks, particularly at relatively low concentrations (<0.1 g L^–1^) and are suitable for spray coating to form thin films on a range of substrates. The extinction spectra of both inks (Figure 1B) show the characteristic π–π* transition peak at ∼300 nm, usually observed for graphene, along with the plateau at higher wavelengths.^27^ The EE ink has a significantly increased peak extinction relative to the plateau value, which implies the EE nanosheets to be thinner than the LPE flakes.^28^ This is consistent with expectations and is because ion intercalation leads to more effective exfoliation during the EE process.^29,30^

**Figure 1:**
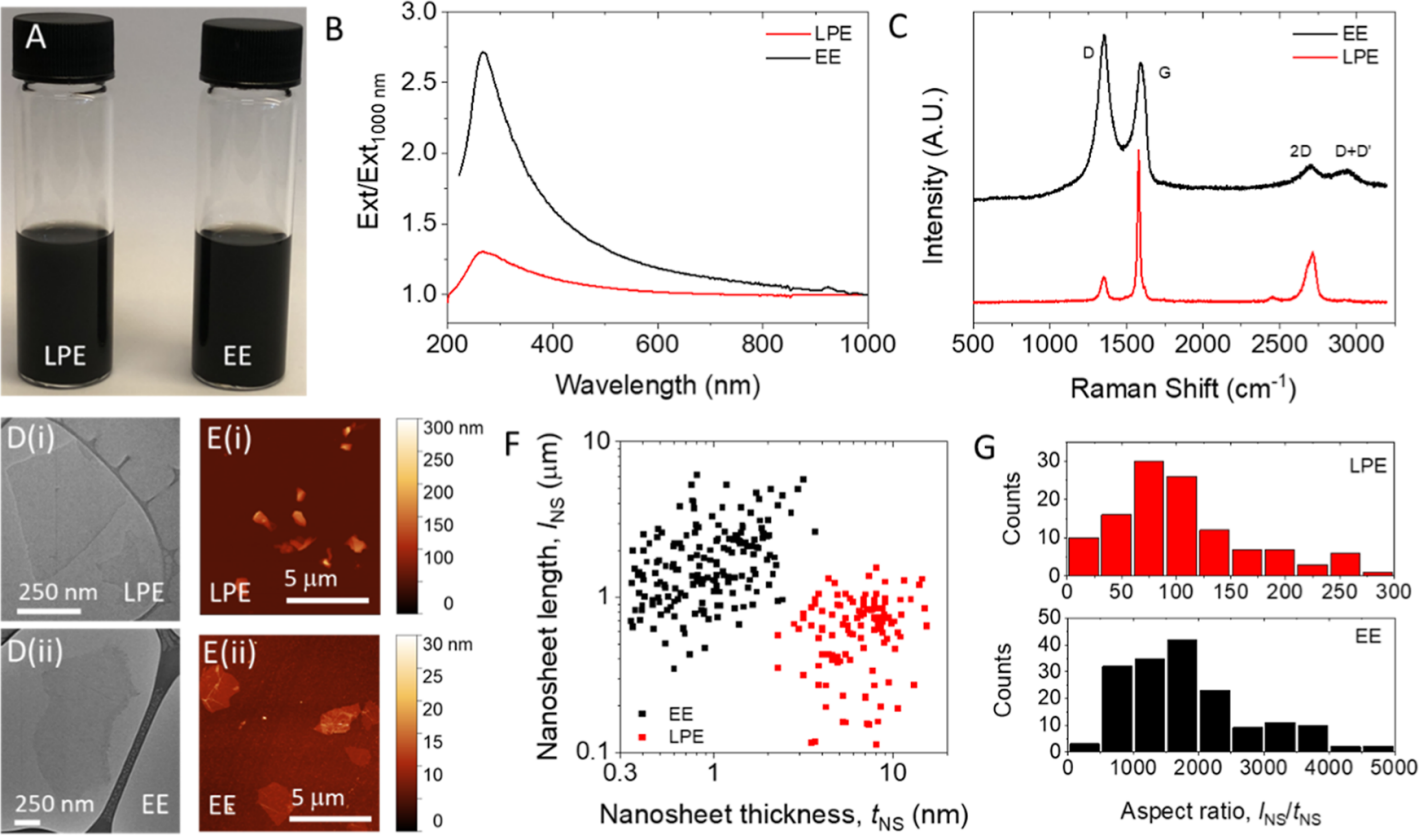
Graphene ink and nanosheet characterization. (A) Photograph of LPE and EE graphene inks dispersed in IPA. (B) UV–vis extinction spectra of LPE and EE nanosheet dispersions. In each case, the extinction is normalized to that at 1000 nm. (C) Raman spectra of LPE and EE films, with an excitation wavelength of 532 nm, showing the characteristic peaks. (D) Representative TEM images of (i) LPE and (ii) EE nanosheets. (E) Representative AFM images of (i) LPE and (ii) EE nanosheets. (F) Plot of nanosheet length vs nanosheet thickness for both nanosheet types. Each data point is an individual nanosheet measured using AFM for both EE (*n* = 180) and LPE (*n* = 120) graphene, respectively. (G) Histograms showing the aspect ratios of LPE (top) and EE (bottom) nanosheets. This data is extracted from that in F.

Raman spectra of the nanosheet films reveal differences in the local crystallinity of the EE and LPE nanosheets (Figure 1C). Most notable is the increased relative intensity amplitude of the D peak (*I*_D_) with respect to the G peak (*I*_G_) in the EE graphene nanosheets compared to the LPE material. The *I*_D_/*I*_G_ ratio is associated with defect content in the nanosheets^31^ and has been measured to be 1.19 and 0.19 for EE and LPE graphene produced in this study, respectively. Greater defect content in EE graphene compared to LPE nanosheets has been previously reported for graphene exfoliated electrochemically using SO_4_^2–^ intercalation.^32^ This likely arises as a result of oxygen covalently bonding to the carbon atoms during exfoliation.^33^

Transmission electron micrographs (Figure 1D) show that the flakes in both inks are well-exfoliated 2D objects. Here, the size difference between nanosheet types is clearly visible, with the EE material having significantly larger lateral sizes when compared to the LPE nanosheets. Representative AFM images for each ink are shown in Figure 1E. From images such as these the length, *l*_NS_, width, *w*_NS_, and thickness, *t*_NS_, of >100 nanosheets were measured for each of the exfoliation techniques. We define the nanosheet length as the longest lateral dimension. In addition, the nanosheet width (*w*_NS_) is the dimension perpendicular to the length. We note that where accurate thicknesses are required, it is important to correct the measured nanosheet thicknesses using an internal calibration like step height analysis.^34–38^ This procedure is described in the Methods. These data are presented as a plot of nanosheet length versus thickness, measured on individual nanosheets as shown in Figure 1F. This graph shows clear differences in both nanosheet length and thickness for EE and LPE nanosheets, with no significant overlap between the two data clouds. This indicates that the two preparation methods yield nanosheets with clearly different size distributions. The mean nanosheet length and thickness for both materials are given in Table 1. Of particular importance is the significant difference in aspect ratio, *k*_NS_ = *l*_NS_/*t*_NS_, between the two materials, which is calculated for each individual nanosheet shown in Figure 1F. This data is plotted as histograms in Figure 1G with the mean aspect ratios given in Table 1. This clearly demonstrates that the LPE and EE techniques produce nanosheets with aspect ratios that are markedly different. It is reasonable to expect that this difference in *k*_NS_ will have a significant impact on the morphology of networks deposited from these two nanosheet types.

**Table 1:** Mean Nanosheet Dimensions from AFM Measurements^a^

	EE graphene	LPE graphene
(A) Measured on Stock Ink Samples e.g. Using Data in Figure 1F		
nanosheet thickness, *t*_NS_	1.08 ± 0.05 nm	7.0 ± 0.3 nm
nanosheet length, *l*_NS_	1875 ± 80 nm	660 ± 30 nm
nanosheet aspect ratio, *k*_NS_	2083 ± 100	108 ± 6
(B) Extracted from Data for Size-Selected Samples as Illustrated in Figure 6A,B		
length: thickness ratio, *k*_lt_	2633 ± 430	104 ± 10
width: thickness ratio, *k*_wt_	1771 ± 290	62 ± 8
length: width ratio, *k*_lw_	1.49 ± 0.01	1.42 ± 0.09

a (A) Average size information measured on the stock EE and LPE graphene inks. Uncertainties are standard errors of the mean. Each value was found by averaging over the sets of values for *t*_NS_, *l*_NS_ and *k*_NS_, measured on individual nanosheets. Nanosheets such as these were studied in Figures 1–3 and 5. (B) Figure 6 focuses on size-selected nanosheets. The aspect ratios extracted from data for these size-selected nanosheets, is shown here. Included are the ratios relating nanosheet length, width, and thickness for EE and LPE graphene. These values were obtained by first finding the mean nanosheet length, width and thickness for each fraction and plotting these parameters versus each other in pairs. Then, the aspect ratios *k*_lt_, *k*_wt_ and *k*_lw_ were obtained from the slopes of graphs of ⟨*l*_NS_⟩ versus ⟨*t*_NS_⟩, ⟨*w*_NS_⟩ versus ⟨*t*_NS_⟩ and ⟨*l*_NS_⟩ versus ⟨*w*_NS_⟩ respectively (see Supporting Information). We note that a slightly different statistical analysis gave very similar aspect ratios (see Supporting Information).

### Basic Film Characterization

Solution-processed 2D inks can be used to produce nanosheet networks using a range of deposition techniques. In this work, spray coating was used due to its procedural simplicity. It is expected that the significant differences in nanosheet aspect ratio shown in Table 1 will result in EE and LPE nanosheets forming films with considerably different morphologies. To assess the nanostructure of both film types, we performed standard SEM imaging followed by 3D imaging.

Figure 2A,B shows representative cross-sectional SEM images of the LPE (A) and EE (B) nanosheet films spray-coated onto glass substrates. From these images, significant morphological differences between the two networks can be observed. The LPE graphene network is clearly very porous with the nanosheets showing poor alignment with the substrate. However, the EE network is relatively featureless in comparison. This implies reduced porosity and increased alignment compared to the LPE network. This is in line with expectations, as reduced porosity has been reported for networks of high aspect ratio nanosheets,^9,13,17^ compared to low aspect ratio LPE material.^39^ As shown below, these structural changes are likely driven by the increased conformability of the larger aspect ratio EE nanosheets (*k*_NS_ = 2083) when compared to their LPE counterparts (*k*_NS_ = 108).

**Figure 2:**
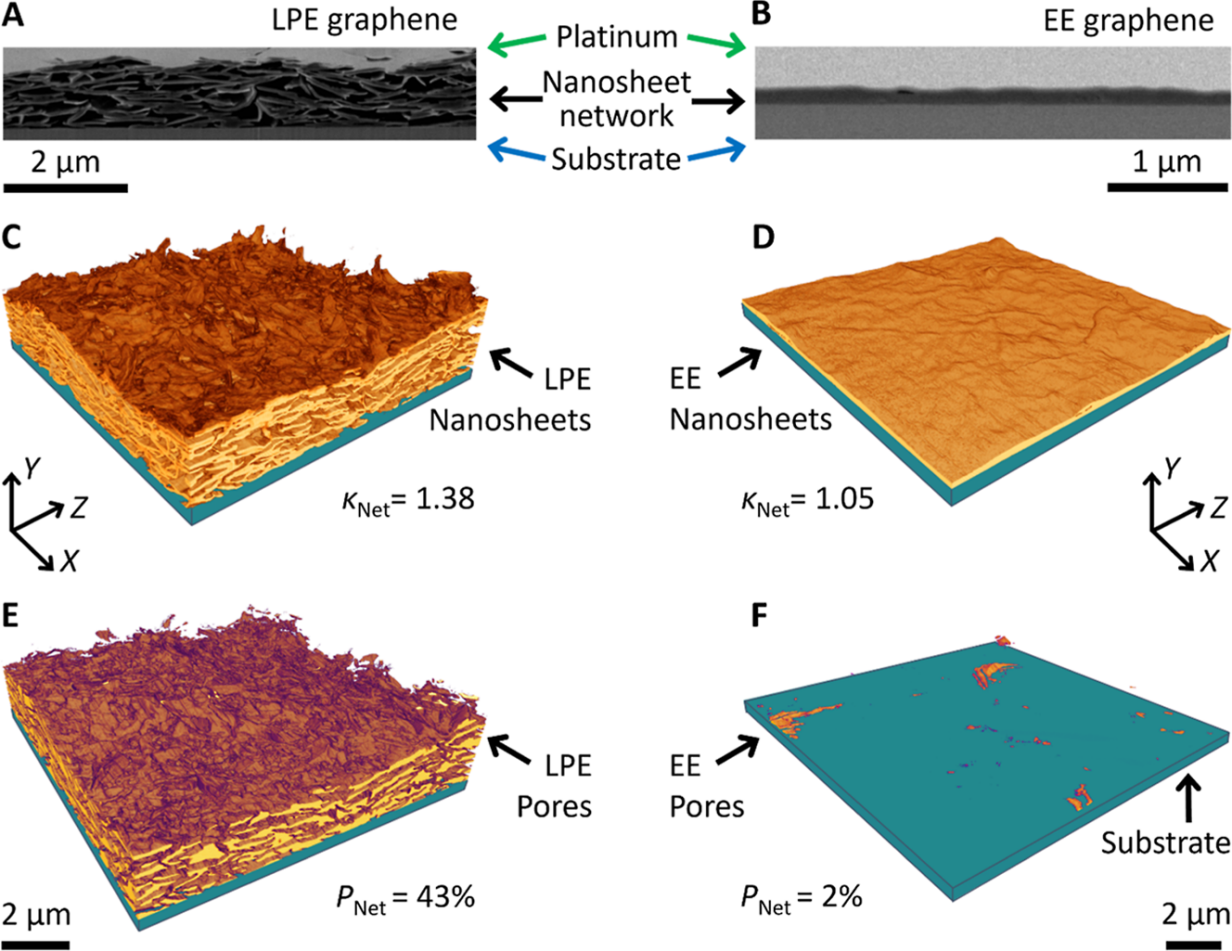
Microscopic network characterization. (A,B) Cross-sectional SEM images of networks produced by spray-coating graphene nanosheets produced by LPE (A) and EE (B). (C,D) 3D reconstructions of the nanosheet volumes for the LPE (C) and EE (D) graphene networks generated using FIB-SEM nanotomography. The in-plane tortuosity factor, κ_Net_, is given for both networks. (E,F) Corresponding images of the pore volumes for LPE (E) and EE (F) graphene networks. The network porosity, *P*_Net_, is given for both networks. 3D images were generated from stacks of successive SEM cross sections from the LPE (*n* = 601) and EE (*n* = 759) networks.

### 3D Imaging

As outlined previously,^40^ FIB-SEM nanotomography can be used to sequentially mill and image a stack of high-resolution network cross sections, such as those shown in Figure 2A,B. Each image in the stack is then segmented into its pore and nanosheet contributions for quantitative analysis. These segmented images can be stitched together to produce high-resolution 3D reconstructions of the networks, allowing their internal nanostructure to be analyzed with a voxel size of ∼5 × 5 × 15 nm.^40^ Shown in Figure 2C,D are sections of such 3D images showing the network of nanosheets for our LPE (Figure 2C) and EE (Figure 2D) networks, respectively. Alternatively, one can reconstruct the internal pore networks as shown in Figure 2E,F. From these 3D images, it is clear that the film of LPE nanosheets (Figure 2C) forms a highly disordered, jammed network characterized by misaligned nanosheets with approximately point-like junctions. This results in considerable porosity as illustrated in Figure 2E. In contrast, the network of EE nanosheets (Figure 2D) appears almost perfectly monolithic. As a result, there are very few pores present inside the network (Figure 2F), which appear very sparse and disconnected.

Such 3D images enable quantification of the network morphology via various parameters including the porosity (fractional pore volume), *P*_Net_, and the tortuosity factor, κ_Net_, of the nanosheet network. Here, κ_Net_ effectively describes how convoluted a path through the nanosheet network is due to the presence of pores. The tortuosity factor has been described as quantifying the deviation of the length of a real transport path, *L*, from the straight-line distance across a network, *L*_0_, such that $\kappa_{Net}={\left(L/L_{0}\right)}^{2}$ , while also accounting for constrictions in the network structure.^41,42^ A tortuosity factor of κ_Net_ = 1 represents a direct path through a monolithic nanosheet network, and values κ_Net_ > 1 indicate a less-well-connected network. For the LPE network, the network porosity was measured to be *P*_Net_ = 43 ± 1%, while the in-plane tortuosity factor was κ_Net_ = 1.38 ± 0.02. However, for the EE nanosheet network, the highly aligned nature of the nanosheets led to a significantly reduced measurable mesoporosity of 2 ± 1% and a much lower tortuosity factor of 1.05 ± 0.02, a value which approaches the lower limit of 1.

Perhaps the clearest difference between the networks is in the degree of nanosheet alignment. To illustrate this, we extracted the root-mean-square roughness, *S*_q_, of the top surface of each network from the 3D images, finding values of *S*_q_ = 24 ± 1 nm and *S*_q_ = 126 ± 3 nm for EE and LPE networks, respectively. The comparatively smaller *S*_q_ value for the EE film is consistent with the reconstructed network volumes in Figure 2C,D, where the EE nanosheets are clearly more well-aligned in the plane of the substrate compared to the LPE nanosheets.

We can quantify this alignment using surface gradient maps of the EE and LPE networks, where the top surface of each network was split into equally sized tiles (330 × 330 nm) for orientation analysis (Figure 3A,B). Each surface tile was approximated as a 2D plane using least-squares fitting, and the polar angle between its normal vector and the *Y*-axis, ϕ_Surf_, was used to determine its orientation (see Supporting Information). A surface tile where ϕ_Surf_ = 0° has a normal vector parallel to the out-of-plane *Y*-direction, meaning the surface is perfectly flat. Surface gradient maps showing ϕ_Surf_ for each tile in the LPE and EE networks are presented in Figure 3A,B and show the LPE network surface to be far more disordered. This can be quantified by plotting histograms of ϕ_Surf_ for each tile in both networks (Figure 3C,D). Here, the LPE graphene network exhibits a broad distribution of surface angles in the range of 0–61°, with a mean value of ${\left\langle \phi_{Surf}\right\rangle }_{LPE}=15.2\pm 0.3^{{}^{\circ}}$ (Figure 3C). In contrast, the surface angle data for the EE network (Figure 3D) displays a much narrower distribution, with a significantly lower mean value of ${\left\langle \phi_{Surf}\right\rangle }_{EE}=3.16\pm 0.06^{{}^{\circ}}$ . Taken together, the data in Figure 3A–D suggests that the nanosheets on the surface of the EE graphene network are much more well-aligned and provide a flatter, more homogeneous interface than their LPE counterparts.

**Figure 3:**
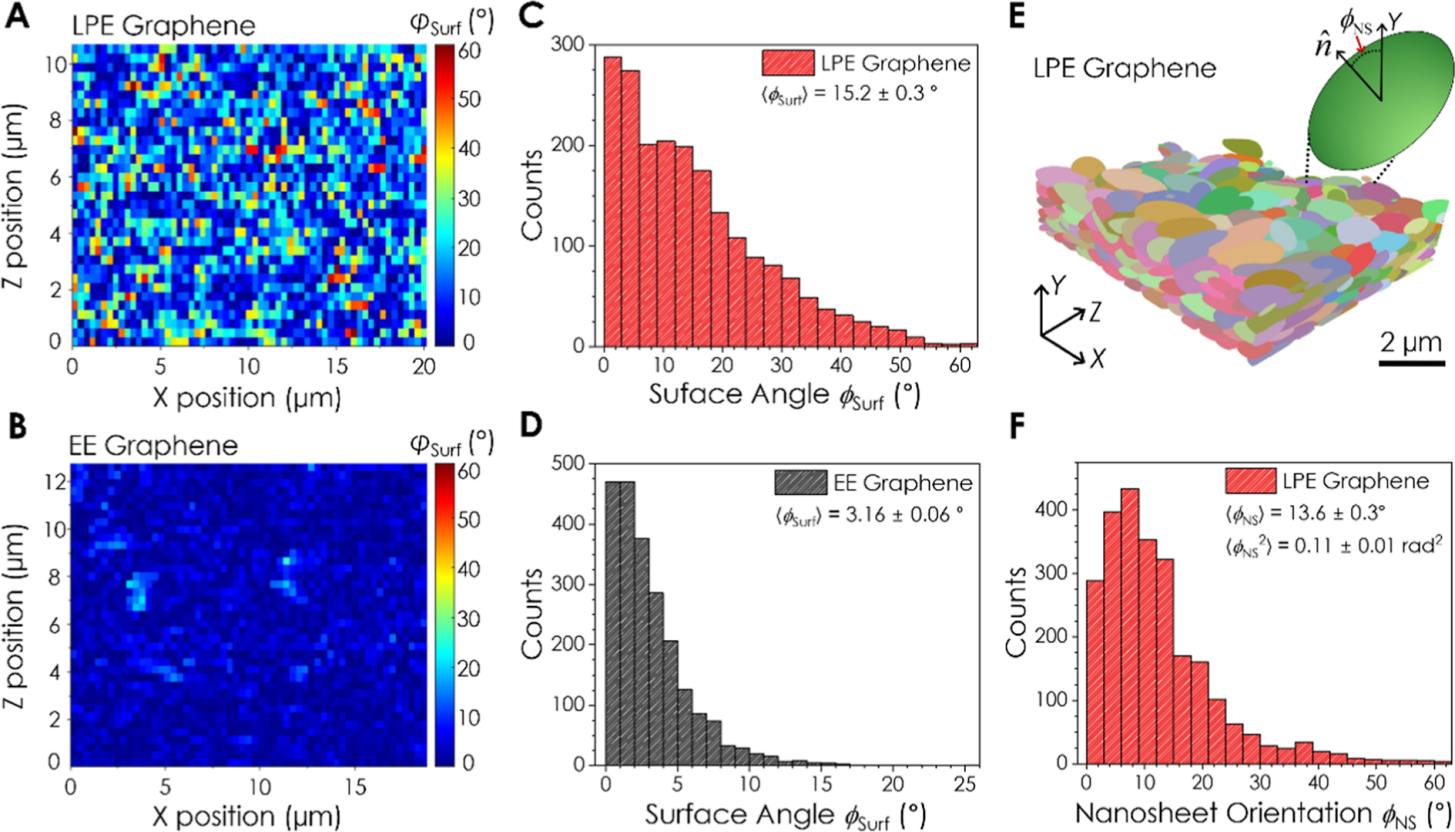
Nanosheet orientation and network surface analysis. (A,B) Surface gradient maps for the LPE (A) and EE (B) graphene networks. The network surfaces were split into discrete tiles and the angle between the normal vector describing each surface tile and the out-of-plane *Y*-direction is given by ϕ_Surf_. When ϕ_Surf_ = 0° the tile describing the network surface is perfectly flat, while larger ϕ_Surf_ values suggest increased disorder. (C,D) Histograms of ϕ_Surf_ values for the LPE (C) and EE (D) graphene networks. The average surface angles for the LPE and EE graphene networks were found to be ⟨ϕ_Surf_⟩ = 15.2 ± 0.3° (*n* = 2016) and ⟨ϕ_Surf_⟩ = 3.16 ± 0.06° (*n* = 2226), respectively. (E) 3D reconstruction of a portion of the LPE graphene network where discrete nanoplatelets have been replaced with equivalent ellipsoids for orientation analysis. Inset: schematic of an ellipsoid, showing the angle, ϕ_NS_, between a nanosheet normal vector, n̂, and the out-of-plane *Y*-direction. When ϕ_NS_ = 0° the nanosheet normal vector is parallel to the *Y*-axis and the nanosheet is perfectly flat. (F) Histogram of ϕ_NS_ values for each of the nanosheets in the LPE graphene network shown in (E). The average angle ⟨ϕ_NS_⟩ and mean squared angle ⟨ϕ_NS_^2^⟩ for nanosheets in the network are given (*n* = 2538).

To probe the nanosheet alignment within the LPE graphene network, we isolated discrete nanoplatelets and fitted equivalent ellipsoids to each (see Supporting Information),^40^ as shown in Figure 3E. Here, the eigenvector describing the ellipsoid’s minor axis can be approximated as the normal vector to the nanosheet, n̂ . The polar angle between n̂ and the out-of-plane *Y*-direction, ϕ_NS_, describes the nanosheet orientation inside the network (Figure 3E). When ϕ_NS_ = 0° the nanosheet is perfectly horizontal. A histogram of the measured ϕ_NS_ values for the LPE graphene network is shown in Figure 3F. Bootstrap analysis was used to ensure that the orientation values extracted from a sample size of *n* ∼ 2500 nanosheets were statistically robust and representative of the global network (Supporting Information Note 4). This data exhibits a broad range of nanosheet orientations with a mean value of ${\left\langle \phi_{NS}\right\rangle }_{LPE}=13.6\pm 0.3^{{}^{\circ}}$ and a mean squared angle of ${\left\langle \phi_{NS}^{2}\right\rangle }_{LPE}=0.11\pm 0.01\;{rad}^{2}$ , with the latter value becoming important below. The value for ${\left\langle \phi_{NS}\right\rangle }_{LPE}$ shows good agreement with the surface alignment data ( ${\left\langle \phi_{Surf}\right\rangle }_{LPE}$ ) for the same network in Figure 3C, indicating that the network surface reflects the orientation distribution of the nanosheets in the bulk of the network.

The conformal tiling of nanosheets in the EE graphene network leads to a monolithic structure (Figure 2D), meaning isolated nanosheets cannot be identified for analysis using this technique of ellipse fitting. However, given the similarity of the mean bulk and surface polar angles for the LPE network (i.e., ${\left\langle \phi_{NS}\right\rangle }_{LPE}$ and ${\left\langle \phi_{Surf}\right\rangle }_{LPE}$ ), we expect the value of ${\left\langle \phi_{Surf}\right\rangle }_{EE}=3.16\pm 0.06^{{}^{\circ}}$ to be a reasonable approximation of the average orientation associated with EE nanosheets in the bulk of the network.

These morphological parameters imply that the networks of EE nanosheets have fewer pores with increased nanosheet alignment and connectivity compared to the LPE networks. This suggests EE nanosheets to be in more intimate contact, likely leading to more large-area, conformal junctions between the sheets. However, it is worth considering why networks of EE nanosheets contain nanosheets which are more aligned than their LPE counterparts.

### The Role of Mechanics in Determining Network Morphology

It is clear from the 3D imaging data that the LPE nanosheets tend to remain rigid, rather than bending, on network formation. Thus, they form a jammed system with significant misalignment and many pores between the nanosheets. This situation is shown schematically in Figure 4A. The monolithic nature of the EE graphene network (Figure 2B) suggests that these nanosheets can effectively conform to their neighbors, thus eliminating porosity. However, in the absence of perfect tiling of the nanosheets into well-ordered layers, such alignment will require the ability of some of the nanosheets to bend in a manner similar to that illustrated in Figure 4B. Clearly, the nature of the internanosheet junctions will be different in these two scenarios with the resultant differences in junction resistance expected to have serious impacts on network conductivity and resistivity.

**Figure 4:**
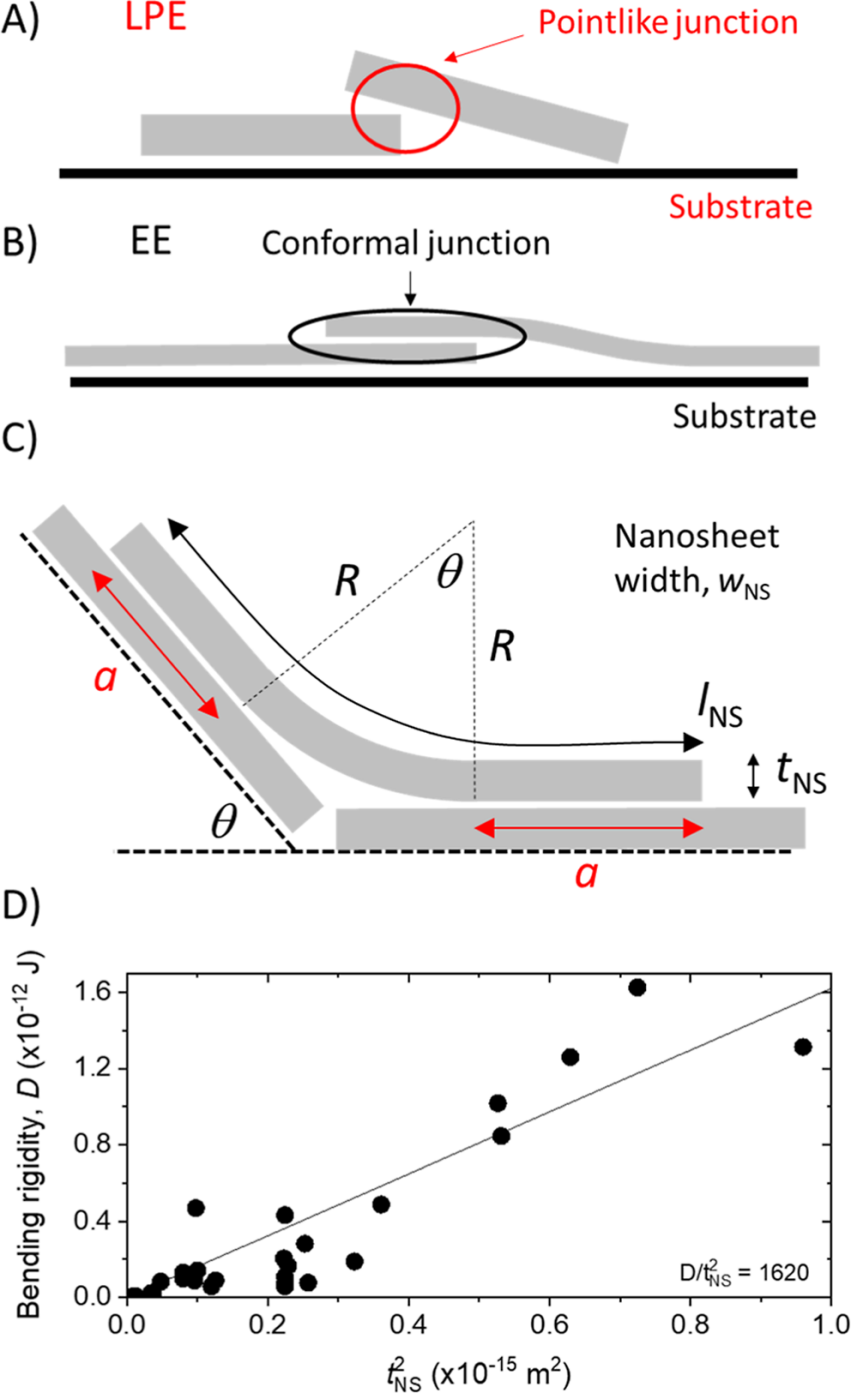
Schematic showing simple models for the relative arrangement of adjacent nanosheets with different aspect ratios. (A) Two relatively rigid, low-aspect-ratio nanosheets, similar to those produced using LPE, which form a point-like or line-like low-area junction. (B) Two high-aspect-ratio nanosheets, similar those produced using EE, which are flexible enough to bend in order to produce a conformal, large-area junction. (C) Diagram showing one nanosheet (top) bending to conform to two other nanosheets (below) which are at an angle θ to each other. The dimensions of the top nanosheet are *l*_NS_, *w*_NS_, *t*_NS_. Also shown are the radius of curvature, *R*, and angle, θ, associated with the bend in the upper nanosheet. The length of the unbent sections at each end of the upper nanosheet are both *a*. (D) Data extracted from ref 48 showing the bending rigidity of graphene sheets plotted versus the square of sheet thickness, *t*_NS_^2^. The straight line has a slope of 1620 J m^–2^.

The scenario shown in Figure 4B involves conformal alignment of one nanosheet to another and will result in relatively large-area and hence low resistance junctions. Thus, it is important to understand under which circumstances this scenario will occur. To analyze this, we consider the situation in Figure 4C. The diagram shows a nanosheet (length, *l*_NS_, width, *w*_NS_, thickness, *t*_NS_) conforming to a surface consisting of two nanosheets at an angle θ to each other. The upper nanosheet is partially adhered to the nanosheets below via van der Waals interactions at each interface. The length of the adhered regions at each end of the nanosheet is *a*, as indicated by the red arrows. In the middle, the upper nanosheet curves through the bend associated with the angle, θ. The radius of curvature of the bend is *R*. Generating this bend costs energy. The only way to pay that energetic cost is via the van der Waals interaction energy between the adjacent nanosheets over the two regions of adherence (each of length *a* and width *w*_NS_).

The equilibrium situation involves a balance of the (negative) energy associated with the adhesion of the nanosheet to the surface and the (positive) energy associated with the curvature of the nanosheet. Modifying the model published by Han et al.,^43^ the total energy includes contributions from the adhesion (first term) and the curvature (second term) such that

$$E=-2w_{NS}a\gamma +\int \;\frac{D}{R^{2}}dA$$

where γ is the energy of adhesion in J m^–2^, *D* is the bending rigidity in Joules, *R* is the radius of curvature in meters and d*A* represents an infinitesimal portion of area of the curved surface in m^2^. The width of the curved region is *w*_NS_, while its length is *R*θ such that *l*_NS_ = 2*a* + *R*θ. This allows us to rewrite the energy as

$$E=-2w_{NS}a\gamma +\frac{D}{R^{2}}w_{NS}R\theta =-w_{NS}\gamma \left(l_{NS}-R\theta \right)+\frac{D}{R}w_{NS}\theta$$

We can find the equilibrium radius of curvature (*R*_0_) from where d*E*/d*R* = 0. Differentiating and then setting d*E*/d*R* = 0 gives $R_{0}=\sqrt{D/\gamma }$ . The equilibrium energy is therefore given by

$$E_{0}=w_{NS}\left(-\gamma l_{NS}+2\theta \sqrt{\gamma D}\right)$$

When *E*_0_ > 0, then this arrangement is no longer stable, and conformation cannot occur. Thus, conformation is impossible when $-\gamma l_{NS}+2\theta \sqrt{\gamma D}>0$ , or when *D* > γ*l*_NS_^2^/4θ^2^. Rearranging, shows us that the conformal junctions shown in Figure 4C can only occur when *l*_NS_^2^ > 4θ^2^ *D*/γ. This is intuitively sensible as it shows that the stiffer the material, the longer the nanosheets need to be to generate conformal junctions.

However, for all materials the bending rigidity increases with thickness. For nanosheets, *D* tends to increase with thickness, *t*_NS_, at a rate intermediate between the monolithic (∼*t*_NS_^3^)^44,45^ and stacked (∼*t*_NS_)^43^ limits, reportedly consistent with *D* = α*t*_NS_^2^,^47^ where α = d*D*/d*t*_NS_^2^ is a material constant. We have extracted data from the literature^48^ for the bending rigidity of graphene nanosheets, which we plot versus *t*_NS_^2^ in Figure 4D. Despite considerable scatter, we find reasonable linearity consistent with α = 1620 ± 170 J m^–2^. Combining this empirical relation with the inequality above yields α*t*_NS_^2^ < γ*l*_NS_^2^/4θ^2^. Rearranging shows that conformal junctions can only occur for nanosheets with aspect ratios above some critical value, i.e. when $l_{NS}/t_{NS}>{\left(l_{NS}/t_{NS}\right)}_{Crit}$ , where

1 $${\left(\frac{l_{NS}}{t_{NS}}\right)}_{Crit}=\sqrt{\frac{4\theta^{2}}{\gamma }\frac{dD}{dt_{NS}^{2}}}$$

­(here we write 2 $\alpha =dD/dt_{NS}^{2}$ ).

Combining this analysis with the 3D imaging results shown in Figure 2 implies that the LPE nanosheets clearly have aspect ratios below the critical value (because they are unbent) while the EE nanosheets must have aspect ratios above the critical value (because the monolithic nature of the network suggests conformal alignment of nanosheets). We can test this numerically for the LPE network, for which it is clear that $l_{NS}/t_{NS}<{\left(l_{NS}/t_{NS}\right)}_{Crit}$ . To do this, we need to know θ, the typical angle over which nanosheets bend. We note that Figure 4C shows a nanosheet bending to conform to both a nanosheet perfectly aligned in plane and one at an angle θ to the horizontal. Inspection of this geometry shows θ to be identical to the orientation angle, ϕ_NS_, between the surface normal of the nanosheet and the vertical direction (Figure 3E). Thus, we can approximate θ^2^ as the mean square orientation angle, ⟨ϕ_NS_^2^⟩, which we can estimate from Figure 3F to be ⟨ϕ_NS_^2^⟩ ∼ 0.11 rad^2^ (i.e., $\sqrt{\left\langle \phi_{NS}^{2}\right\rangle }\approx 19^{{}^{\circ}}$ ) for LPE nanosheets. Then taking the value of α given above and approximating γ as the surface energy of LPE graphene (40 mJ m^–2^)^35^ yields ${\left(l_{NS}/t_{NS}\right)}_{Crit}∼$ 130. Even though this analysis is quite crude, it is consistent with the data as Figure 1G clearly shows the majority of LPE nanosheets to have aspect ratios <130. This clearly links the nanosheet aspect ratio to the observed morphology. In addition, we expect this morphology and its associated junction types to have a significant impact on the electrical properties of the nanosheet networks.

### Dependence of Electrical Properties of LPE and EE Nanosheets on Network Thickness

In this section we will discuss the effect of network thickness (*t*_Net_) on the network resistivity. First it is worth noting that, like most particulate films, nanosheet networks display disorder. One manifestation of this disorder is the presence of spatial variations in network thickness. Such variations are unavoidable and can be observed in even the most ordered solution-deposited nanosheet films.^49^ The fractional thickness variation tends to be relatively small in thicker films but increases with decreasing thickness.^49^ In very thin films these thickness variations can lead to significant spatial non uniformity which is of course related to the percolation effects described below. In this work, the thicknesses of our thinnest films are measured optically which means they are spatially averaged over large areas (∼cm^2^). However, in these very thin networks, the local thickness can deviate significantly from the mean.

It is well-known that the electrical conductivity of nanosheet^11,23,50–52^ and nanoparticle^53–56^ thin films depends on network thickness. As the thickness of the network, *t*_Net_, increases, electrical conduction through the network undergoes a percolative transition from insulating, when there are no connected pathways, to a percolating regime where the number of pathways increases with thickness. In this regime, the conductivity increases with network thickness to a bulk-like intrinsic conduction state which is characterized by a conductivity that is independent of network thickness.^51^ The onset of conduction occurs at a specific thickness known as the percolation threshold, *t*_c_, while the transition from percolative to bulk-like conduction occurs at a second specific thickness which we label *t*_*x*_.

When the film is very thin and *t*_Net_ < *t*_c_, there is no connected pathway between the electrodes and so the substrate is visible, with isolated nanosheets scattered on the surface or in clusters smaller than the interelectrode separation. This is observed for both EE and LPE graphene films in Figure 5A,C. As the film thickness increases, the gaps between the nanosheets are filled in with additional material, increasing the surface coverage and network conductivity until no substrate is observed in the top-down SEM images of the bulk networks where *t*_Net_ > *t*_*x*_ (Figure 5B,D). Interestingly, similar fill-in processes occur for both systems as *t*_Net_ is increased, despite the differences in nanosheet aspect ratio and network morphologies.

**Figure 5:**
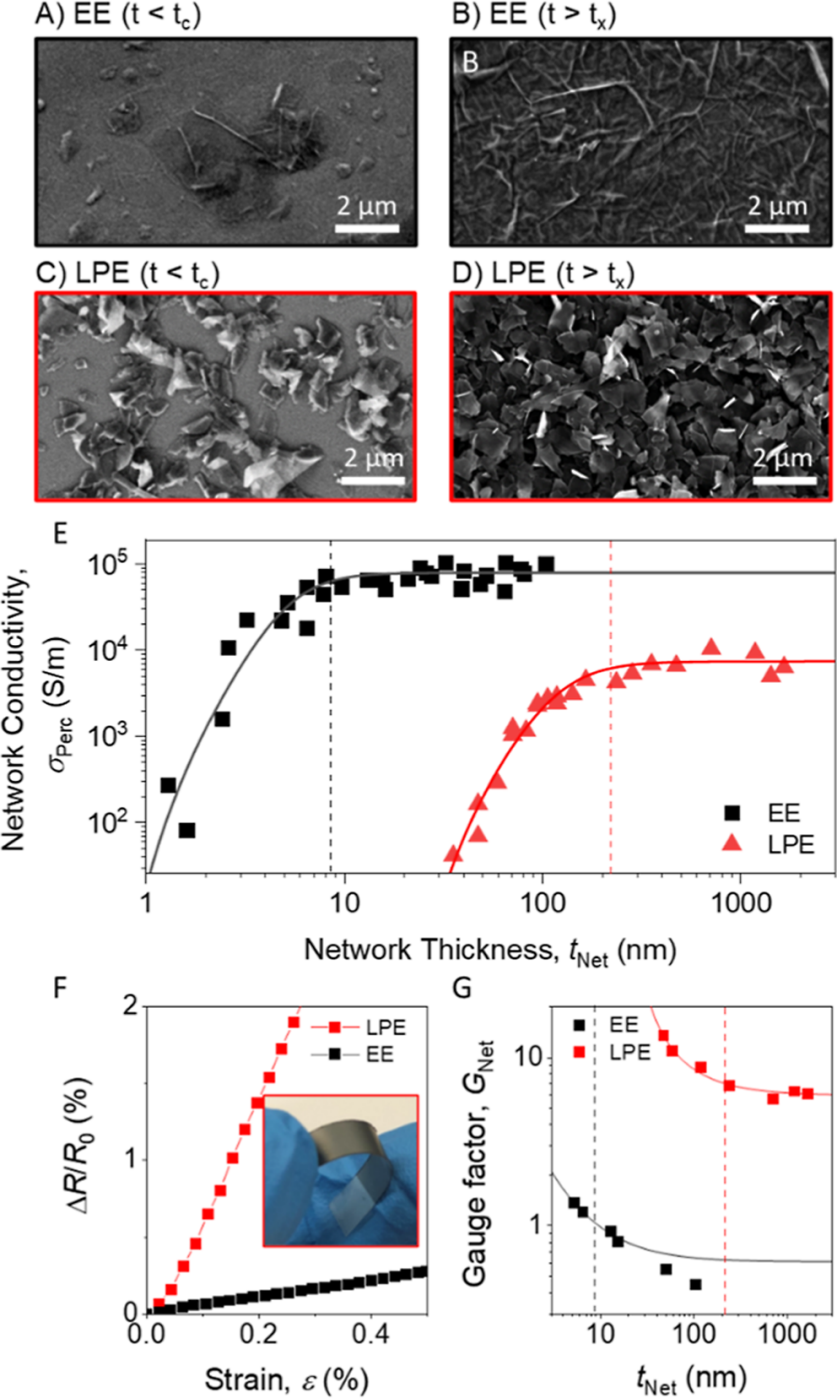
Electrical characterization of LPE and EE graphene nanosheet networks. (A–D) SEM images of the top surface of spray-coated networks for both nanosheet types, each at two network thicknesses, *t*_Net_. (A,B) Show EE nanosheet networks while (C,D) show LPE nanosheet networks. However, (A,C) show extremely thin networks while (B,D) show relatively thick networks. A: *t*_Net_ < 1 nm; B: *t*_Net_ > 10 nm; C: *t*_Net_ < 30 nm; D: *t*_Net_ > 200 nm. (E) Plot of network conductivity vs network thickness for EE and LPE nanosheet films. The solid lines are fits to the percolation model given in eq 2. Fit parameters are shown in Table 2. (F) Plot of the fractional change in resistance, Δ*R*/*R*_0_, vs strain, ε, for bulk-like networks prepared with LPE (*t*_Net_ = 237 nm) and EE (*t*_Net_ = 50 nm) nanosheets at a strain rate of 0.2% s^–1^. Inset: Photograph of a sprayed film of LPE nanosheets on PET, bent between two fingers. (G) Plot of gauge factor vs network thickness for EE and LPE graphene. The solid lines represent fits of eq 3 to the data in the percolation regime. In (E,G) the vertical black and red dashed lines represent the values of *t*_*x*_ for the EE and LPE networks, respectively.

To quantify this transition, samples with a range of network thicknesses were spray-coated for both the LPE and EE graphene inks and their electrical conductivity was measured. Values of *t*_Net_ were chosen to span from below the percolation thickness, *t*_c_, through the percolative region where conductivity scales with thickness, and up to thicknesses where the network conductivity reaches a plateau of bulk-like values, as shown in Figure 5E.

To analyze this data, we use a semiempirical model which describes the electrical conductivity in both the percolative and bulk-like thickness regimes^57^

2 $$\sigma_{Perc}\approx \sigma_{Net}{\left[1+\frac{1}{4}{\left(\frac{t_{x}-t_{c}}{t_{Net}-t_{c}}\right)}^{n}\right]}^{-1}$$

This equation is essentially a manifestation of Matthiessen’s rule which, in its most general form, states that when more than one source of resistance is present, the total resistance is the sum of the various contributions. Here, eq 2 sums resistivity in the bulk-like regime with a percolative term. In this equation, σ_Perc_ represents the thickness-dependent network conductivity while σ_Net_ represents the thickness-independent conductivity of the network in the bulk-like regime. In addition, *n* is the percolation exponent. We note that σ_Perc_ → σ_Net_ when *t*_Net_ > *t*_*x*_. We can think of σ_Net_ as an intrinsic property of networks, which are thick enough to eliminate all percolative effects. This model enables key network parameters, including σ_Net_ and *t*_*x*_ to be extracted.

Above the bulk transition thickness, *t*_*x*_, the bulk-like conductivity of LPE and EE nanosheet networks is estimated to be σ_Net_ = 7349 S m^–1^ and 78,550 S m^–1^, respectively, by averaging over the data in the plateau region. Similar conductivity values were reported for LPE graphene networks (8000 S m^–1^) by Gabbett & Doolan *et al.* ^40^ and for EE graphene networks (86,800 S m^–1^) by Marković *et al*.^19^

We then fixed the values of σ_Net_ and fit the data to eq 2 to find the remaining parameters. The extracted values from fitting are given in Table 2. The percolation exponents for the EE networks (*n* = 3.1 ± 1.0) and LPE networks (*n* = 2.7 ± 0.9) were in line with previous values for spray-cast graphene networks.^11^ We note that percolation exponents higher than the theoretically predicted value of 2 are expected in the presence of a broad distribution of junction resistances within the network.^58^

**Table 2:** Values Extracted from Fitting the Electrical and Piezoresistive Response of EE and LPE Graphene Films in Figure 5 as a Function of Network Thickness^a^

	EE graphene networks	LPE graphene networks
σ_Net_ (fixed)	78,550 ± 5050 S m^–1^	7349 ± 820 S m^–1^
*t*_c_	0.7 ± 0.5 nm	20 ± 8 nm
*t*_*x*_	8.6 ± 2.0 nm	207 ± 32 nm
*n*	3.1 ± 1.0	2.7 ± 0.9
*G*_TNS_	0.61 ± 0.04	5.9 ± 0.2
*t*_TNS_	3.5 ± 0.3 nm	206 ± 14 nm

a For conductivity versus thickness fitting (Figure 5E), σ_Net_ was fixed at a value found by averaging the highest conductivity LPE (n = 6) and EE (*n* = 13) data points (σ_Net_ error is standard error of the mean). In practice the log of the conductivity data was fitted using the log of eq 2. For gauge factor versus thickness fitting (Figure 5G), *t*_c_ was fixed at the values found during conductivity versus thickness fitting for each nanosheet type.

Here, it is observed that the thinner, more conformal EE nanosheets begin to form conductive pathways at low network thicknesses of *t*_c_ ∼ 0.7 ± 0.5 nm, compared to the LPE nanosheet network which displays *t*_c_ ∼ 20 ± 5 nm. This is probably due to the better in-plane alignment of the EE nanosheets as well as the fact that they have higher aspect ratios. Similar observations can be made regarding the bulk-like transition point, *t*_*x*_, in both cases. This transition occurs at 8.6 ± 2.0 nm and 207 ± 32 nm for the EE and LPE networks, respectively. These differences are again related to nanosheet alignment as well as nanosheet thickness.^51^ The ability to produce bulk-like networks at lower film thicknesses is desirable for printed electronic applications. For example, printed transistors based on semiconducting nanosheets display mobilities that increase with network thickness.^15,59^ However, screening effects mean that the on/off ratio is such devices tends to degrade with increasing network thickness.^59,60^ This means one can simultaneously obtain better combinations of mobility and on/off ratio in those networks which become bulk-like at lower thickness. This suggests a particular advantage to using high aspect ratio nanosheets in such applications.

### Piezoresistive Response

We can directly illustrate how morphology effects network applications by considering piezoresistance, the physical effect underpinning strain sensors. Piezoresistance describes the change in electrical resistance of a film due to mechanical deformation, quantified by the gauge factor, which we refer to as *G*_Net_. This parameter is defined as the fractional resistance change per unit strain: $G_{Net}=\lim_{\varepsilon \to o}\left[\Delta R/R_{0}\right]/\varepsilon$ .^61^ To assess the influence of nanosheet aspect ratio and network morphology on *G*_Net_, thick networks (*t*_Net_ > *t*_*x*_) of LPE and EE graphene nanosheets were spray-coated onto PET strips for piezoresistive testing (Figure 5F). The resultant fractional resistance change, (Δ*R*/*R*_0_), is plotted as a function of strain, ε, for both network types in Figure 5F. The LPE network demonstrated a significantly higher piezoresistive response (*G*_Net_ ∼ 7) compared to the EE network (*G*_Net_ ∼ 0.6). This is attributed to nanosheets in the EE network sliding freely while maintaining conformal contact, whereas LPE nanosheets exhibit point-like contacts that separate under strain, increasing the measured resistance. This work is consistent with previous reports which show that higher conductivity networks tend to display reduced gauge factors.^12,62–65^

To study this in more detail, we spray-coated films of varying thickness for each nanosheet type and measured *G*_Net_. As shown in Figure 5G, in both cases we found a clear increase in *G*_Net_ with decreasing *t*_Net_. It has been shown that, for *t*_Net_ < *t*_*x*_, the gauge factor of percolative networks is described by

3 $$G_{Net}\approx G_{TNS}+\frac{t_{TNS}}{t_{Net}-t_{c}}$$

where *t*_c_ is the percolation threshold, while *G*_TNS_ and *t*_TNS_ are figures of merit that both have their origin in network morphological effects.^11^ For both materials, fits were performed while fixing *t*_c_ at the values found above (Table 2), with fitting yielding values for *G*_TNS_ and *t*_TNS_ (see Table 2). We found that, while the LPE network data were well-described by this model, it only fit the EE network data at the lowest network thicknesses. This might imply that different piezoresistive mechanisms are at work in EE networks. In any case, it is clear that the piezoresistive response is much larger for the LPE networks than for the EE films. As larger values of these parameters are desirable if these networks are to be used in strain sensors, this shows that LPE nanosheets are superior for such an application. This is a clear example of morphological effects strongly influencing parameters that control material performance in applications.

### Nanosheet Size-Dependent Conduction in EE and LPE Networks

Above we have discussed the conductivity of nanosheet networks, obtaining for example the bulk-like conductivity, σ_Net_. However, below we will focus on the network resistivity, ρ_Net_ (σ_Net_ = 1/ρ_Net_). This is simply because the model we will apply has a simpler nanosheet size dependence when applied to resistivity rather than conductivity. It has recently been shown that the bulk-like resistivity of networks of nanosheets with high carrier density (such as graphene) is given by^14^

4 $$\rho_{Net}\approx \frac{\kappa_{Net}}{\left(1-P_{Net}\right)}\left[\rho_{NS}+2t_{NS}R_{J}\right]$$

where κ_Net_ and *P*_Net_ are the network tortuosity factor and porosity, ρ_NS_ is the resistivity of the individual nanosheets, *R*_J_ is the junction resistance and *t*_NS_ is the nanosheet thickness. This relationship means that one can obtain information about the conduction process (e.g., ρ_NS_ and *R*_J_) by measuring the resistivity of networks fabricated from nanosheets of different thicknesses.

To achieve this, LPE and EE nanosheet inks were size-selected using liquid cascade centrifugation.^66^ For each nanosheet type, this resulted in six fractions, each containing nanosheets with different sizes. For all fractions, we performed AFM to measure the distribution of nanosheet lengths, widths, and thicknesses. As an example, the flake-by-flake measurements of nanosheet length versus thickness are plotted for the largest and smallest size-selected fractions for the LPE and EE nanosheets in Figure 6A, (see Supporting Information for all data). In both cases, we found the largest fraction to have considerably longer and thicker nanosheets relative to the smaller fraction. This is consistent with observations for liquid exfoliated nanosheets, which show the larger nanosheets to be proportionately thicker.^67^ For each fraction, we plot the average nanosheet length and thickness as a function of the centrifugation speed used during each size selection step (Figure 6B). This graph clearly shows that for all fractions the EE nanosheets were both longer and thinner than the LPE nanosheets. It also clearly shows the average nanosheet length and average nanosheet thickness to scale with centrifugation speed (rpm) in the same way, consistent with them being roughly proportional. Such proportionality is predicted by simple models^67^ and supported by various published data.^14,38,67–70^ We can quantify this scaling for our EE and LPE nanosheets by defining average aspect ratios relating the nanosheet length, width, and thickness as *k*_lt_ = *l*_NS_/*t*_NS_, *k*_wt_ = *w*_NS_/*t*_NS_ and *k*_lw_ = *l*_NS_/*w*_NS_ (note that we have changed the notation relative to above to differentiate the aspect ratios of the size-selected samples discussed here from the nonsize-selected samples discussed above). The mean nanosheet aspect ratios for each material type were determined from AFM measurements (see Supporting Information) and are given in Table 1B. For illustration, we have plotted the relevant mean *k*_lt_ aspect ratios in Figure 6A, as dashed lines.

**Figure 6:**
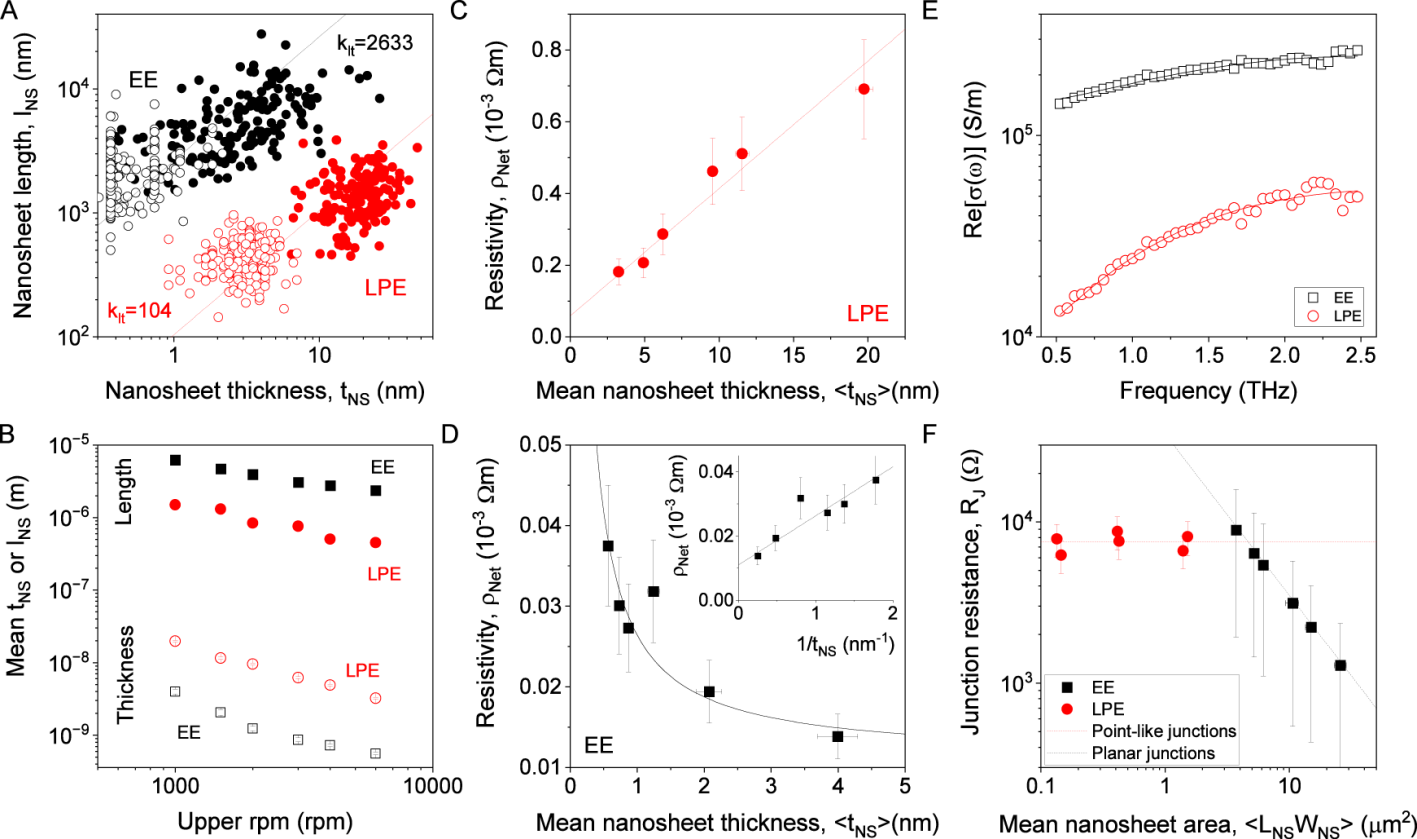
Characterization of size-selected graphene nanosheets and their networks. (A) Plots of nanosheet length vs nanosheet thickness for LPE (red) and EE (black) graphene nanosheets as measured by AFM. This panel shows data collected from the largest and smallest fractions where each data point represents an individual nanosheet. (B) Mean nanosheet lengths and thicknesses plotted versus the upper centrifugation speed used in the size-selection procedure for both EE and LPE nanosheets. (C,D) Network resistivity plotted versus mean nanosheet thickness for networks of LPE (C) and EE nanosheets (D). The solid lines in C and D are fits to eqs 4 and 6 respectively. The inset shows the EE data linearized as per eq 6. (E) Data for the real part of the THz conductivity measured versus frequency for both LPE and EE nanosheets. The lines are fits to the Drude–Smith model. (F) Junction resistances, extracted from the data in D&E plotted versus the mean nanosheet area for each size. The red and black dashed lines illustrate the expected behavior for point-like and planar junctions, respectively.

We spray-coated inks containing each size fraction into networks for both nanosheet types. In all cases the networks had thicknesses *t*_Net_ > *t*_*x*_ to ensure they are in the bulk-like regime, with *t*_Net_ in the range of 1–3 μm for LPE nanosheets and *t*_Net_ ranging from 30 to 160 nm for EE nanosheets. For each network we measured the (bulk-like) DC resistivity, ρ_Net_. This is plotted as a function of nanosheet thickness for LPE nanosheets in Figure 6C. The data is consistent with eq 4, which predicts that ρ_Net_ scales linearly with *t*_NS_, with the associated fit shown as the solid line. Combining this fit with the values of κ_Net_ = 1.38 and *P*_Net_ = 0.43 measured above (Figure 2), we can find the nanosheet resistivity, ρ_NS_ = (2.4 ± 1.1) × 10^–5^ Ω m and junction resistance, *R*_J_ = 7340 ± 1030 Ω. These values are in line with previous estimates for LPE graphene exfoliated in NMP (ρ_NS_ = (2.9 ± 1.3) × 10^–5^ Ω m and *R*_J_ = 8900 ± 1000 Ω) and water–surfactant solutions (ρ_NS_ = 1.7 × 10^–5^ Ω m and *R*_J_ = 3300 Ω).^14,71^

The resistivity values for networks of EE nanosheets are plotted as a function of nanosheet thickness in Figure 6D. This graph shows ρ_Net_ to fall with increasing *t*_NS_, and so appears to be inconsistent with eq 4. However, this is not the case. As discussed above, networks of EE nanosheets are highly aligned and are expected to have conformal, large-area junctions. Assuming the junction area is proportional to nanosheet area, then larger nanosheets with have bigger junctions, leading to lower junction resistances and decreased network resistivity.

We can express these ideas quantitatively as follows. For aligned, conformal junctions with large junction area, we expect: *R*_J_ = (*RA*)_J_/*A*_J_, where (*RA*)_J_ is a parameter describing intersheet charge transport and *A*_J_ is the junction area. Assuming the junction area is a fixed fraction (*f*_J_) of the nanosheet area (*A*_NS_, which we approximate as the nanosheet length times width), we have *A*_J_ = *f*_J_ *A*_NS_ = *f*_J_ *l*_NS_ *w*_NS_. This means the resistance of a planar junction is given by

5a $$R_{J}=\frac{{\left(RA\right)}_{J}}{f_{J}l_{NS}w_{NS}}$$

At this point, it must be noted that, as described above, liquid exfoliated nanosheets tend to have well-defined relationships between thickness and both length and width. Then, relating *l*_NS_ and *w*_NS_ to *t*_NS_ via the aspect ratios defined above allows us to write the junction resistance in terms of nanosheet thickness

5b $$R_{J}=\frac{{\left(RA\right)}_{J}}{f_{J}k_{lt}k_{wt}t_{NS}^{2}}$$

Inserting eq 5b into [Disp-formula eq4] gives

6 $$\rho_{Net}\approx \frac{\kappa_{Net}}{\left(1-P_{Net}\right)}\left[\rho_{NS}+\frac{2{\left(RA\right)}_{J}}{f_{J}k_{lt}k_{wt}}\frac{1}{t_{NS}}\right]$$

which is appropriate for networks of high aspect ratio EE nanosheets (but not low aspect ratio LPE nanosheets). Crucially, this equation has a different size scaling to eq 4 and predicts that ρ_Net_ scales linearly with 1/*t*_NS_, which we find to be the case in Figure 6E. We fit this data with eq 6, finding very good agreement. Combining the fit parameters with measured values for κ_Net_ = 1.05, *P*_Net_ = 0.02, *k*_lt_ = 2633 and *k*_wt_ = 1771 will allow us to obtain ρ_NS_ and (*RA*)_J_, once we know *f*_J_. Previously we have measured *f*_J_ in networks of EE MoS_2_ using SEM imaging, finding values of 0.33,^16^ and 0.4.^14^ Assuming EE graphene behaves similarly, we can estimate *f*_J_ = 0.37 ± 0.03. This allows us to calculate ρ_NS_ = (1.0 ± 0.3) × 10^–5^ Ω m and (*RA*)_J_ = (1.2 ± 0.8) × 10^–8^ Ω m^2^.

### Understanding Nanosheet Resistivity Data

We first consider the nanosheet resistivity. The values of ρ_NS_ = (2.4 ± 1.3) × 10^–5^ Ω m for LPE networks and (1.0 ± 0.3) × 10^–5^ Ω m for EE networks, extracted via the network model are in line with measured in-plane resistivities for graphite which are in the range ∼10^–5^ to 10^–6^ Ω m.^72^ What is slightly surprising is that the resistivity of the EE nanosheets is lower than that of the LPE nanosheets given the high defect content of the former (see Figure 1C).

To test this unexpected result, we performed terahertz spectroscopy to probe the intrinsic electrical properties of the nanosheets making up the networks.^21,73,74^ To do this, we measured the frequency dependent conductivity on printed nanosheet networks for both EE and LPE graphene. Due to the very high driving frequency, THz radiation induces motion of carriers over very short distances, resulting in only local, and so intrananosheet, charge transport. The real part of the terahertz conductivity is plotted versus frequency in Figure 6E for both materials. Such data can be fit using the real part of the Drude–Smith equation^75,76^

7 $$Re\left[\sigma \left(\omega \right)\right]=\frac{\sigma_{NS}}{1+\omega^{2}\tau_{NS}^{2}}\left[1+c_{NS}\frac{\left(1-\omega^{2}\tau_{NS}^{2}\right)}{\left(1+\omega^{2}\tau_{NS}^{2}\right)}\right]$$

here, σ_NS_ is the nanosheet conductivity (σ_NS_ = 1/ρ_NS_), τ_NS_ is the scattering time while *c*_NS_ is the backscattering parameter (varying between 0 and −1 indicating a range from isotropic scattering to complete backscattering). From these data, we can extract the nanosheet mobility (μ_NS_) and carrier density (*n*_NS_).

For both materials, eq 7 fits the data well, yielding ρ_NS_ values of (8.8 ± 0.3) × 10^–6^ Ω m and (2.1 ± 0.1) × 10^–6^ Ω m for LPE and EE nanosheets, respectively. Both values are lower than their DC counterparts extracted from the model, due to the relatively short length scales probed. However, we do indeed find higher intrinsic resistivity for the LPE nanosheets, consistent with the outputs of the network model.

In addition, the fits outputted τ_NS_ values of 54 ± 4 fs and 51 ± 5 fs and *c*_NS_ values of −0.93 ± 0.02 and −0.72 ± 0.02 for LPE and EE nanosheets, respectively. The *c*_NS_ values indicate that backscattering is predominant in both cases, but slightly less pronounced in the EE case. From these quantities, we can calculate μ_NS_ values which were 1426 ± 253 cm^2^/(V s) and 717 ± 148 cm^2^/(V s) for LPE and EE nanosheets, respectively. Here the higher intrinsic mobility in the LPE sample is consistent with the lower defect content observed by Raman spectroscopy. Finally, the associated carrier densities for LPE and EE nanosheets were (5.0 ± 0.4) × 10^24^ m^–3^ and (4.1 ± 0.2) × 10^25^ m^–3^ respectively. This shows that the lower resistivity displayed by the EE nanosheets is due to their higher carrier density which is probably associated with doping occurring during the exfoliation process.

### Understanding Junction Resistance Data

In order to better understand the junction resistance results, we use the parameters obtained above to calculate *R*_J_ for each size-selected network, for each nanosheet type. For the LPE nanosheets we rearrange eq 4 and use the previously quoted values of ρ_NS_, κ_Net_ and *P*_Net_, to calculate *R*_J_ for each nanosheet thickness and corresponding value of ρ_Net_. For the EE nanosheets, we use eq 5a and the previously quoted values of (*RA*)_J_ and *f*_J_, as well as the measured mean values of *l*_NS_ and *w*_NS_ (see Supporting Information). The resultant *R*_J_ values are plotted versus nanosheet area (*l*_NS_ *w*_NS_) in Figure 6F for both LPE and EE nanosheets.

We find the junction resistance values for the LPE nanosheets to be reasonably independent of nanosheet area at ∼6–7 kΩ. This is consistent with the linearity observed in Figure 6C which implies a single *R*_J_ value for the entire data set. That *R*_J_ is independent of LPE nanosheet area implies that the junctions in LPE networks are point-like (see Supporting Information). If they were linear junctions associated with nanosheet edges, or planar junctions associated with nanosheet surfaces, the junction area would scale with nanosheet size. It is likely that the junctions predominately consist of nanosheet vertices interacting with the basal planes of adjacent nanosheets. However, the area of such “point-like” junctions might be expected to depend on nanosheet thickness. It may be that some deformation occurs at the contact point that yields roughly the same contact area irrespective of nanosheet thickness.

For the networks of EE nanosheets, *R*_J_ scales inversely with nanosheet area as defined by eq 5a. We note that this trend is evidenced by the agreement between experiment and theory in Figure 6F. This behavior has clear practical importance as it means that, as long as the nanosheet aspect ratio is above the critical value, larger area nanosheets will lead to lower junction resistances. This will clearly be important in networks of semiconducting nanosheets where junction resistances are usually large.^14^ In addition, this highlights a significant advantage of using networks of 2D nanosheets over other particle geometries such as 1D nanotubes. While it is possible to increase the junction area by increasing the nanosheet size, this is not practical for nanotube networks where the junction area is limited by the nanotube diameter.

As shown in Figure 6F, in almost all cases, the EE graphene networks have lower junction resistance than the LPE networks. This is a significant factor leading to the consistently lower resistivity measured in EE networks compared to LPE. However, what is surprising is that the *R*_J_ values for the LPE networks, which we believe originate in point like junctions, are similar in magnitude to those of the smallest EE nanosheets. To put this in context, we note that the smallest EE nanosheets in this work have an area of ∼4 μm^2^ and so have junction areas of ∼1.5 μm^2^, assuming *f*_J_ ∼ 0.37. In contrast, a nanosheet which is ∼10 nm thick should only form a point-like junction of area ∼100s of nm^2^ (∼10^–4^ μm^2^). Even an edge-to-plane junction would only have an area of ∼10^–2^ μm^2^ for a nanosheet with *l*_NS_ ∼ 1 μm and *t*_NS_ ∼ 10 nm. Thus, we would expect low-aspect ratio (LPE) graphene nanosheets to have much larger *R*_J_ than high aspect ratio (EE) ones, as is observed for semiconducting nanosheet networks.^14^

There are likely two explanations for this incongruity. First, it is probable that not all of the area of planar junctions is active for internanosheet charge transport. There is almost certainly organic residue (residual solvent etc.) trapped in the junction that limits the area where internanosheet hopping can occur.^77^ This means that (*RA*)_J_ is probably far below its ideal value. We can crudely illustrate this as follows. Consider a planar junction between two nanosheets where the stacking at the interface between the nanosheets is perfect (i.e., AB stacking). This allows us to model the entire junction region as a slab of graphite. Then, the junction resistance is just the out-of-plane resistance of a piece of graphite with the same thickness as the nanosheet and the same area as the junction. Then *R*_J_ = ρ_⊥_ *t*_NS_/*A*_J_ where ρ_⊥_ is the out-of-plane resistivity of the graphite. This means that in the ideal case: (*RA*)_J_ = ρ_⊥_ *t*_NS_. For graphite, ρ_⊥_ ∼ 10^–3^ Ω m,^72^ and taking *t*_NS_ ∼ 1 nm, yields a minimal value of (*RA*)_J_ = 10^–12^ Ω m^2^, almost 4 orders of magnitude below the value found above. Of course, real junctions are not perfect so this discrepancy has many causes, including the presence of organic residue, imperfect stacking, misalignment etc., nevertheless it shows that junction resistance can be reduced significantly from what is currently observed. We note that these factors above have not been well-studied in the context of internanosheet junctions. In the future, it will be important that a rigorous experimental exploration of these effects is carried out.

A second possible cause may be that internanosheet charge transfer to or from a portion of an edge may be inherently more likely than plane-to-plane transfer. Indeed, studies have shown that electrochemical electron transfer from graphene edges is roughly × 100 faster than that from the basal plane.^78^ If a similar effect is present for internanosheet charge transfer, it might explain why the junction resistances observed for LPE nanosheets are not considerably higher as might be expected from their small contact area.

### Insights into Network Transport

Finally, we can use these learnings to gain further insights to why networks of EE nanosheets are less resistive than LPE-based networks. It is possible to rearrange eq 4 as follows

8 $$\frac{\rho_{Net}}{\rho_{NS}}\approx \frac{\kappa_{Net}}{\left(1-P_{Net}\right)}\left[1+\frac{R_{J}}{R_{NS}}\right]$$

where we use the fact that *R*_NS_ = ρ_NS_/2*t*_NS_, where the factor of 2 comes from the fact that in a network, current passes on average through half the length of each nanosheet.^14^ This means that aside from the tortuosity and porosity, the critical factor limiting the network resistivity is *R*_J_/*R*_NS_. Clearly, we want this parameter minimized such that *R*_J_ ≪ *R*_NS_. However, *R*_NS_ always depends on nanosheet thickness while *R*_J_ is either constant (low aspect ratio) or depends on junction area (via eq 5a, high aspect ratio).

To understand the factors limiting conduction in networks, we plot *R*_J_ and *R*_NS_ versus *t*_NS_ in Figure 7A,B for high-aspect-ratio (EE) and low-aspect-ratio (LPE) nanosheets, respectively. To do this, we used the concepts and parameters quoted above. For both high- and low-aspect ratio nanosheets, *R*_NS_ falls with increasing *t*_NS_ as *R*_NS_ = ρ_NS_/2*t*_NS_. For low aspect ratio nanosheets, *R*_J_ is constant (see Figure 6F) consistent with point-like junctions. However, for the high-aspect-ratio nanosheets *R*_J_ ∝ 1/*A*_NS_ due to the planar nature of the junctions (eq 5a). For nanosheets with constant aspect ratio, this means *R*_J_ ∝ 1/*t*_NS_^2^ (eq 5b). For ease of discussion, we assume constant aspect ratio in Figure 7A. We find that *R*_J_ falls faster than *R*_NS_ for the high-aspect-ratio nanosheets: thicker nanosheets have *R*_J_ < *R*_NS_ with the opposite being true for thinner nanosheets (Figure 7A). Conversely, because *R*_J_ is constant for low-aspect-ratio nanosheets, only the thinnest nanosheets have *R*_J_ < *R*_NS_ (Figure 7B).

**Figure 7:**
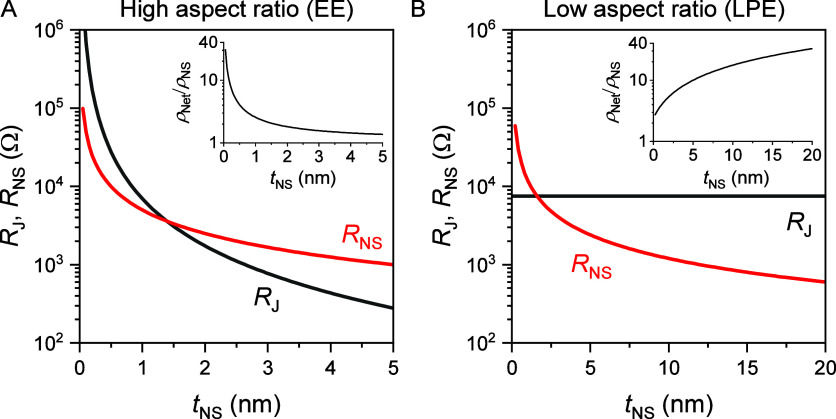
Understanding how network resistivity depends on nanosheet thickness. (A,B) Theoretical plots of both nanosheet, *R*_NS_, and junction, *R*_J_, resistances for networks of (A) high aspect ratio nanosheets, such as those produced using EE, with planar junctions and (B) low aspect ratio nanosheets, such as those produced using LPE, with point-like junctions. The insets plot the resultant network resistivity, normalized to the nanosheet resistivity, ρ_Net_/ρ_NS_, as a function of *t*_NS_. The *R*_J_ curve in (A) was plotted using eq 5b and the parameters: (*RA*)_J_ = 1.2 × 10^–8^ Ω m^2^, *f*_J_ = 0.37, *k*_lt_ = 2,633, *k*_wt_ = 1771. In B, *R*_J_ = 7513 Ω. The *R*_NS_ curves were plotted using *R*_NS_ = ρ_NS_/2*t*_NS_ with ρ_NS_ values of 1 × 10^–5^ Ω m and 2.4 × 10^–5^ Ω m in A and B respectively. All numerical values are consistent with the main text.

We have used these curves to plot the ratio of the network and nanosheet resistivities, ρ_Net_/ρ_NS_, as a function of nanosheet thickness for EE and LPE graphene in the insets of Figure 7A,B. These clearly show that for high-aspect-ratio nanosheets (Figure 7A), the network resistivity approaches its minimum value when the nanosheet thickness gets large enough. This is because when the aspect ratio is both constant and high, thick nanosheets have large area and hence large *A*_J_ which leads to *R*_J_ ≪ *R*_NS_. Conversely, for low aspect ratio nanosheets (Figure 7B), the conditions where *R*_J_ ≪ *R*_NS_ occurs at low *t*_NS_ leading to minimization of ρ_Net_/ρ_NS_ for very thin nanosheets. This is due to the high *R*_NS_ associated with very thin nanosheets.

The latter scenario may explain why networks of very thin, low aspect ratio MoS_2_ nanosheets have been reported to have surprisingly high conductivity (up to 10^–3^ S m^–1^).^79^ The former scenario indirectly explains why networks of monolayer reduced graphene oxide display resistivities as low as 10^–5^ Ω m.^80^ Although GO nanosheets do not have fixed aspect ratios, they are very thin and have large areas and so large *A*_J_, a combination which leads to small *R*_J_/*R*_NS_ and low resistivity.

## Conclusions

This study underscores the pivotal role of nanosheet aspect ratio in dictating the morphology, junction resistance, and electrical properties of solution-processed graphene networks. Through a comparative analysis of liquid-phase exfoliated (LPE) and electrochemically exfoliated (EE) graphene, we reveal that higher aspect ratios facilitate superior nanosheet alignment, leading to lower porosity, increased connectivity, and the formation of large-area, conformal junctions. As a result, EE networks exhibit significantly lower resistivity and higher conductivity compared to LPE networks, largely due to reduced junction resistance.

Furthermore, electrical modeling confirms that while LPE networks display constant junction resistance due to their point-like contacts, EE nanosheet networks display junction resistance which decreases with increasing nanosheet area. This yields much lower junction resistances for large area nanosheets. In addition, because the nanosheet resistance scales inversely with nanosheet thickness, thin nanosheets have high nanosheet resistance. Because minimizing *R*_J_/*R*_NS_ is the most important factor for achieving high network mobility and conductivity, this shows very clearly that networks of large area monolayers^59^ are most desirable when fabricating conductive networks.

Although this work uses graphene as a model system, we believe that the learnings broadly apply to networks of other 2D materials such as MoS_2_, WSe_2_ or InSe. We have previously shown that nanosheet networks of various 2D materials have similar structures^40^ and obey the same conduction rules.^14,81^ Thus, we expect the models and concepts outlined here to be transferrable across different materials. Although different 2D materials will possess different mechanical characteristics, interlayer interaction strengths, resistivities and intersheet junction resistances, this work links the critical aspect ratio and network resistivity to these properties allowing their application to a range of materials.

These findings provide key insights for optimizing solution-processed nanosheet networks in printed electronics, sensors, and flexible devices. By tailoring nanosheet aspect ratio and junction properties, future studies can further enhance the performance of 2D material networks for next-generation electronic applications.

## Methods

### LPE Graphene Exfoliation

Graphite flakes (Asbury, 4 g) were sonicated in 80 mL *n*-methyl-2-pyrollidone (Sigma) for 1 h at 65% power. The resulting suspension was centrifuged (Hettich) at 6000 rpm for 1 h and the sediment was resuspended in 80 mL of *n*-methyl-2-pyrollidone. This was horn sonicated for 9 h at 60% power. This gave the as-exfoliated dispersion. The dispersion was centrifuged for 90 min at 2000 rpm to sediment the unexfoliated graphite and the supernatant was centrifuged for 90 min at 4000 rpm. The sediment was resuspended in fresh IPA (Sigma) and centrifuged for 60 min at 6000 rpm. This washing step was repeated twice. The final sediment was resuspended in IPA and the concentration of the dispersion was determined from UV–vis measurements with metrics from Backes et al.^28^

### EE Graphene Exfoliation

Two pieces of graphite foil (Alfa Aesar, 50 × 30 × 0.254 mm^3^) were used as anode and cathode for the EE process. Both electrodes were immersed in 100 mL of 0.1 M aqueous (NH_4_)_2_SO_4_ with a constant separation of 2 cm, and a 10 V DC voltage was applied for 30 min. The current rises steadily during the process, from ∼1.1–1.5 A to ∼2–2.5 A. The process is repeated using a fresh graphite foil anode, and the expanded material is collected via filtration and repeatedly washed with deionized water (2 L). The expanded material is then transferred to a bottle containing 200 mL of DMF and sonicated for 10 min (Fisherbrand FB11205, 37 kHz, 100% power). This was the as-exfoliated dispersion. To remove nonexfoliated graphite, the dispersion was centrifuged for 20 min at 3000 rpm (Hettich, 10 cm rotor radius) and the supernatant was decanted without redispersion of the sediment. The resulting dispersion was centrifuged for 90 min at 6000 rpm, the supernatant was discarded, and the sediment was redispersed in 20 mL of fresh DMF via brief sonication. The concentration of this ink (∼1.5 mg/mL) was determined via UV–vis measurement.^82^ The ink was solvent exchanged to IPA, via 2 cycles of centrifugation (90 min, 6000 rpm) and redispersed via brief bath sonication immediately before use.

### Size Selection of Nanosheet Dispersions

The liquid cascade centrifugation process was applied to as-exfoliated dispersions (prior to the removal of nonexfoliated material) in order to extract a range of nanosheet sizes for both exfoliation processes. Each as-exfoliated suspension went through the following process. The nanosheet dispersion was centrifuged at 500 rpm at 5 °C for 2 h, the sediment was discarded, and the supernatant was centrifuged at 1000 rpm at 5 °C for 2 h. The sediment from this centrifugation was labeled according to the lower and upper trapping speeds in krpm. The remaining supernatant was centrifuged at 1500 rpm at 5 °C for 2 h, separating the sediment and supernatant, as before. The centrifugation cycles were repeated at 2000, 3000, 4000, and 6000 rpm, in each instance the sediment was kept and labeled according to the trapping speeds used. Once the centrifugation steps were completed, the sedimented material was resuspended in fresh IPA, centrifuged at 6000 rpm at 5 °C for 1 h. These sediments were resuspended for a final time in fresh IPA to yield a set of size selected graphene dispersions.

### Nanosheet Characterization (UV–Vis, Raman, TEM, AFM, SEM)

The nanosheet based inks were characterized using UV–vis spectroscopy (Cary 50). Raman spectra were acquired (using a Renishaw inVia Qontor Raman spectrometer, illuminated by a 532 nm wavelength laser) and averaged over 3 accumulations with a 100× objective of a sample of each ink deposited on glass. An incident power of ∼1 mW was used to minimize possible thermal damage. TEM images of the sheets were collected using a JEOL 2100, with an accelerating voltage of 200 kV, in bright field mode. AFM was conducted, using Digital Instruments Multimode IIIa in tapping mode. Inks were drop cast onto Si/SiO_2_ substrates coated with (3-aminopropyl)­triethoxysilane as reported by Karger et al.^83^ 10 × 10 μm^2^ images were recorded. For solution-processed nanosheet networks, it is important to account for the difference between the measured thickness and the true thickness of the nanosheets by determining the layer number from AFM metrics.^34,66,84–86^ For graphene, each layer has a step height of 0.95 nm, with 1 nm of solvent trapped between the nanosheet and substrate which must also be subtracted. From the layer number, the true nanosheet thickness can be calculated, as each layer has a true thickness of 0.35 nm.^35^ The SEM of the top surfaces of the films were imaged using a (Zeiss) Ultra SEM with a secondary electron detector. The working distance was set to 5 mm, a potential of 3 keV, with an aperture size of 30 μm.

### Substrate Preparation

Glass substrates (Epredia) were cleaned by a 2-step bath sonication in acetone and IPA (5 min in each solvent). The substrates were subsequently dried with compressed N_2_. PET substrates were cleaned using IPA and dried with compressed N_2_.

### Film Deposition

Graphene films were spray coated onto the glass substrates using a modified airbrush (Harder & Steenbeck Infinity Airbrush), mounted in a Janome (JR2300N) mobile gantry. The gantry was programmed to raster across the area of the mask (2 cm × 4 cm), with the serpentine pattern mask attached to the substrate with Kapton tape. The nozzle top substrate working distance was 10 cm and the N_2_ back pressure was set to 3.5 bar. The ink concentration was 0.1 mg/mL for LPE graphene and 0.05 mg/mL for EE graphene, and ink flow was kept constant at 60 mL/h.

### Thickness Characterization

Film thicknesses were determined using a stylus profiler (Veeco Dektak 6m). This had a stylus radius of 12.5 μm, using the force setting at 1 mg, and sampled every 0.2 μm along the profile. For thinner films where stylus profilometry was not suitable, optical profilometry using the Filmetrics Profilm3D with a 50× Nikon objective lens^87^ and flatbed transmission scanning methods^11^ (Epson Perfection V700 PHOTO) were used.

### Thermal Annealing

To remove residual solvents and increase electrical conductivity, graphene films on glass were thermally annealed in a vacuum chamber (VC2509A) at 350 °C for 1 h.

### Electrical Testing

Four probe DC electrical characterization was conducted in ambient conditions by contacting silver paint electrodes with probes connected to an electrical source meter (Keithley KE2612A) operating in a four-probe sense mode.

### THz Spectroscopy

Nonsize-selected inks of both LPE and EE graphene were diluted to 0.05–0.1 mg/mL graphene in IPA. Using a previously described approach,^15^ thin films were made via a liquid–liquid interfacial deposition technique employing deionized water (45 mL, >18 MΩ·cm) and *n*-hexane (15 mL, Sigma-Aldrich; >99%). Quartz substrates were cleaned with acetone and IPA and UV-ozone cleaned for 3 min prior to deposition. Films were deposited on half of each quartz slide by covering one-half with aluminum foil. Four subsequent depositions were done to make a 4 L film of EE-graphene and two subsequent depositions were done to make a 2 L film of LPE-graphene to achieve ∼50% transmission in the films. Films were dried at a slight angle on a hot plate at ∼80 °C in-between depositions. For complete drying, films were annealed at 200 °C under vacuum for 1 h and allowed to cool overnight under vacuum.

Each sample was half covered during deposition such that the uncovered half serves as an on-chip reference, eliminating realignment errors when the optical path was switched between sample and reference. THz transmission measurements were carried out with a THz spectrometer driven by a 90 fs pulsed 1.55 μm, 100 MHz Er-doped fiber laser (Menlo Systems). The laser delivered ≈20 mW average power to both the photoconductive emitter and detector antennas (Menlo Systems). Time resolved THz transients from both sample and the on-chip reference were recorded. To suppress internal Fabry–Perot echoes of the 1.5 mm quartz, a simple time gate was applied that removed the first reflection. Fast Fourier transformation yields the complex transmission spectrum, which was converted to sheet conductivity σ­(ω) with Tinkham’s thin-film equation.^88^ Volume conductivity was obtained by dividing σ by the optical thickness which is estimated from the 650 nm absorption using 2.3% absorption per graphene monolayer. Conductivity spectra were fitted with the Drude–Smith model to extract the Drude conductivity σ_NS_, localization parameter *c*_NS_, and momentum-scattering time τ_NS_. Carrier mobility was calculated under the assumption of *v*_F_ = 1 × 10^6^ m s^–1^ for graphene, using the following equation^89^

$$\mu_{NS}=\frac{e^{3}v_{f}^{2}}{\pi \hbar^{2}}\frac{\tau_{NS}^{2}}{\sigma_{NS}}$$

For both materials, independent measurements were made on two separate spots and the fit parameters averaged.

### Electromechanical Testing

The nanosheet networks were contacted with silver paint contacts and silver wires connected to an electrical source meter (Keithley KE2601) in a two-probe electrical configuration. The resistance was measured simultaneously with the tensile test carried out with a Zwick Z0.5 ProLine Tensile Tester with a 100 N load cell. Devices were tested between 0% and 0.5% strain with a strain rate of 0.2%/s.

### 3D Imaging

A dual beam FIB-SEM microscope (Auriga, Carl ZEISS) was used to mill and image network cross sections of the printed nanosheet networks. All images were captured at a working distance of 5 mm using a 2 kV accelerating voltage and 30 μm aperture. FIB-SEM nanotomography was performed using ATLAS 5 software (version 5.3.3.31) as described in ref 40. Network cross sections were milled using a 30 kV: 600 pA gallium ion beam and all images were captured with a pixel size of 5 nm using both the SE2 and Inlens detectors. Image stacks for the LPE and EE graphene networks were aligned and converted to 3D images using Dragonfly (2022.2.0.1409, Object Research Systems). Network cross sections captured using the SE2 detector were segmented into their discrete nanosheet and pore components using the Trainable WEKA Segmentation^90^ plugin in FIJI.^91^ To assist the image classification, the pixel intensity in each image stack was first normalized, and the image brightness and contrast were tuned to maximize contrast between the nanosheet and pore phases. The network porosity and tortuosity factor were measured using the Taufactor application^92^ in MATLAB. Individual nanoplatelets in the LPE graphene network were identified using a 3D Distance Transform Watershed within the MorphoLibJ plugin^93^ in FIJI. A chessboard distance transform with a dynamic setting of 2 was used. Surface gradient maps and the roughness of the LPE and EE graphene networks were generated in MATLAB. The FIB-SEM nanotomography process and image processing pipeline are described in detail in ref 40.

## Supplementary Material



## Notes

AFM measurements of nanosheet dimensions, surface gradient maps of networks, nanosheet orientation, statistical robustness of orientation data from FIB-SEM-NT generated volumes, and internanosheet junctions in printed LPE graphene networks (PDF)
