# Supplementary material for: Quantifying the Influence of Nanosheet Aspect Ratio on Network Morphology and Junction Resistance in Solution-Processed Nanosheet Networks
Source: ACS Nano. 2025 Sep 11;19(37):33118–33. doi: 10.1021/acsnano.5c04008 (PMC12462246; doi:10.1021/acsnano.5c04008)
Supplement: Supplementary file 1 [file nn5c04008_si_001.pdf]

Supplementary Information for

Quantifying the influence of nanosheet aspect ratio on network morphology and junction resistance in solution-processed nanosheet networks

Eoin Caffrey[†1], Jose M. Munuera[†1,2], Cian Gabbett[1], Luke Doolan[1], Joseph Neilson[1], Rebekah A. Wells,[1] Mark McCrystall[1], Alexandra McNamara[1], Tian Carey[1], Martin Gerlei,[3] Paul Seifert,[3] Georg S. Duesberg,[3] Jonathan N. Coleman[1]*

[1]*School of Physics, CRANN & AMBER Research Centres, Trinity College Dublin, Dublin 2, Ireland*

[2]*Instituto de Ciencia y Tecnología del Carbono, INCAR-CSIC, C/Francisco Pintado Fe 26, 33011 Oviedo, Spain*

[3]*Institute of Physics, University of the Bundeswehr Munich, Werner-Heisenberg-Weg 39, D-85579 Neubiberg, Germany*

*colemaj@tcd.ie (Jonathan N. Coleman); Tel: +353 (0) 1 8963859.

Table of Contents

Supplementary Note 1: AFM Measurements of Nanosheet Dimensions for LPE and EE Graphene 2

Supplementary Note 2: Surface Gradient Maps of LPE and EE Graphene Networks 10

Supplementary Note 3: Nanosheet Orientation within the LPE Graphene Network 13

Supplementary Note 4: Statistical Robustness of Orientation Data from FIB-SEM NT Generated Volumes 15

Supplementary Note 5: Inter-nanosheet Junctions in Printed LPE Graphene Networks 18

Supplementary Note 1: AFM Measurements of Nanosheet Dimensions for LPE and EE Graphene

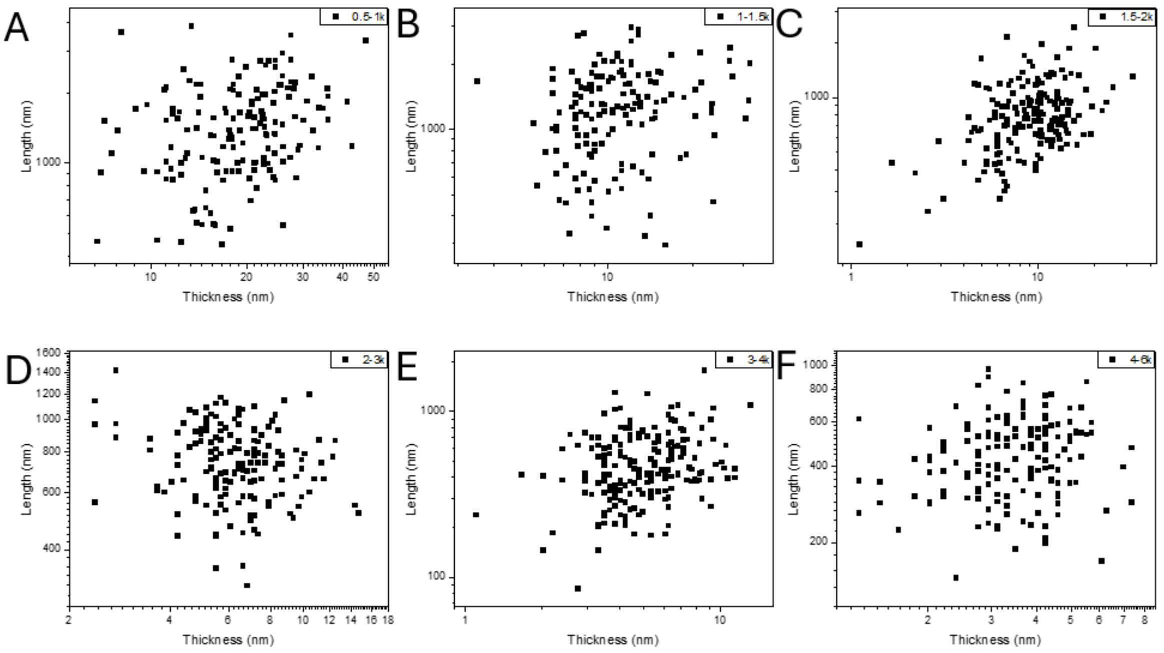

Figure S1: Plot of length vs. thickness (corrected) for individual nanosheets as measured by AFM for six size-selected fractions of LPE graphene. The data in **(A-F)** are sorted by increased centrifugation trapping speed.

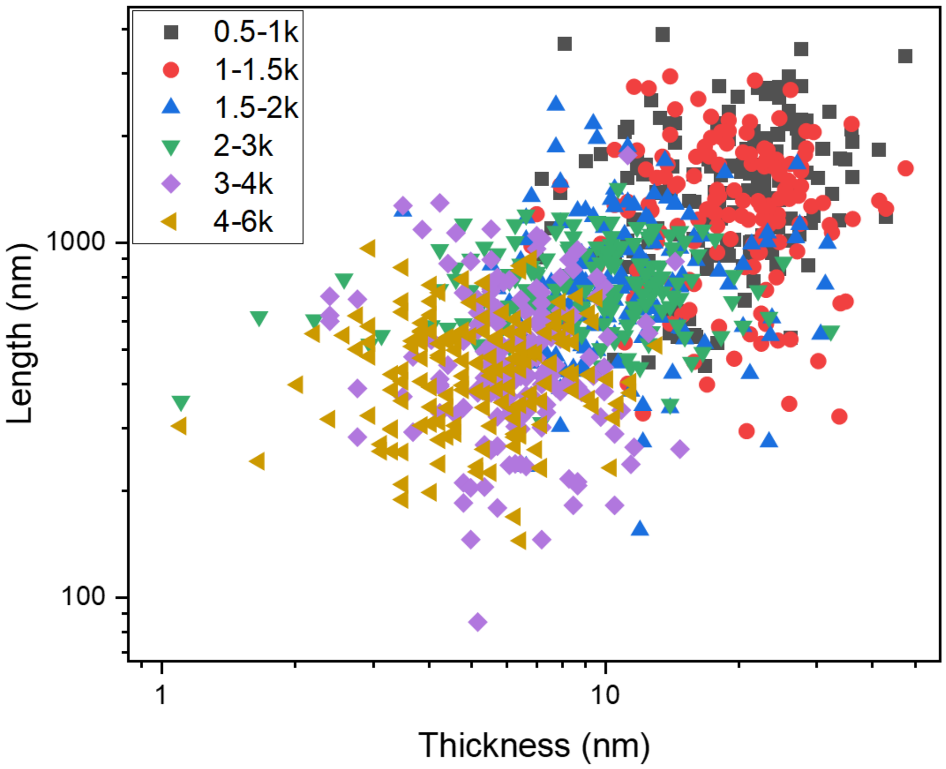

Figure S2: Plot of length vs. thickness (corrected) for individual nanosheets as measured by AFM for six size-selected fractions of LPE graphene. The centrifugation speeds used to trap each size fraction are given in krpm.

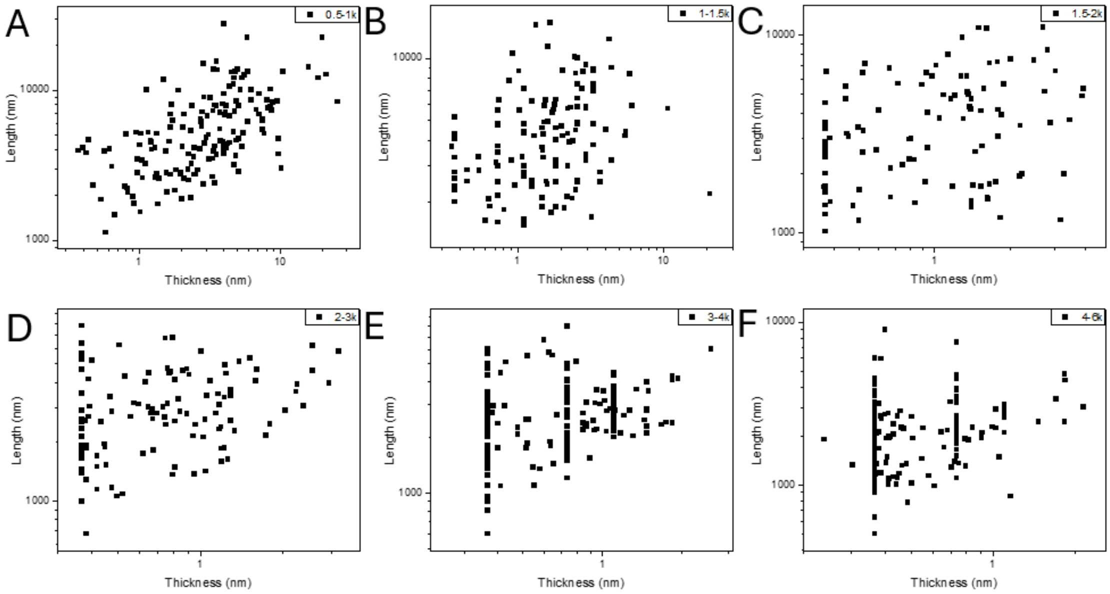

Figure S3: Plot of length vs. thickness (corrected) for individual nanosheets as measured by AFM for six size-selected fractions of EE graphene. The data in **(A-F)** are sorted by increased centrifugation trapping speed.

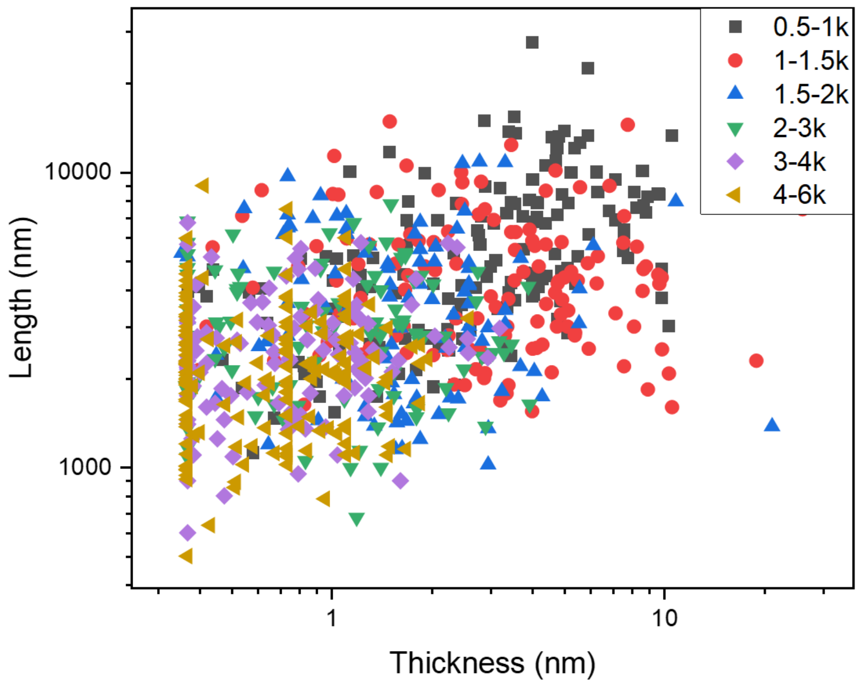

Figure S4: Plot of length vs. thickness (corrected) for individual nanosheets as measured by AFM for six size-selected fractions of EE graphene. The centrifugation speeds used to trap each size fraction are given in krpm.

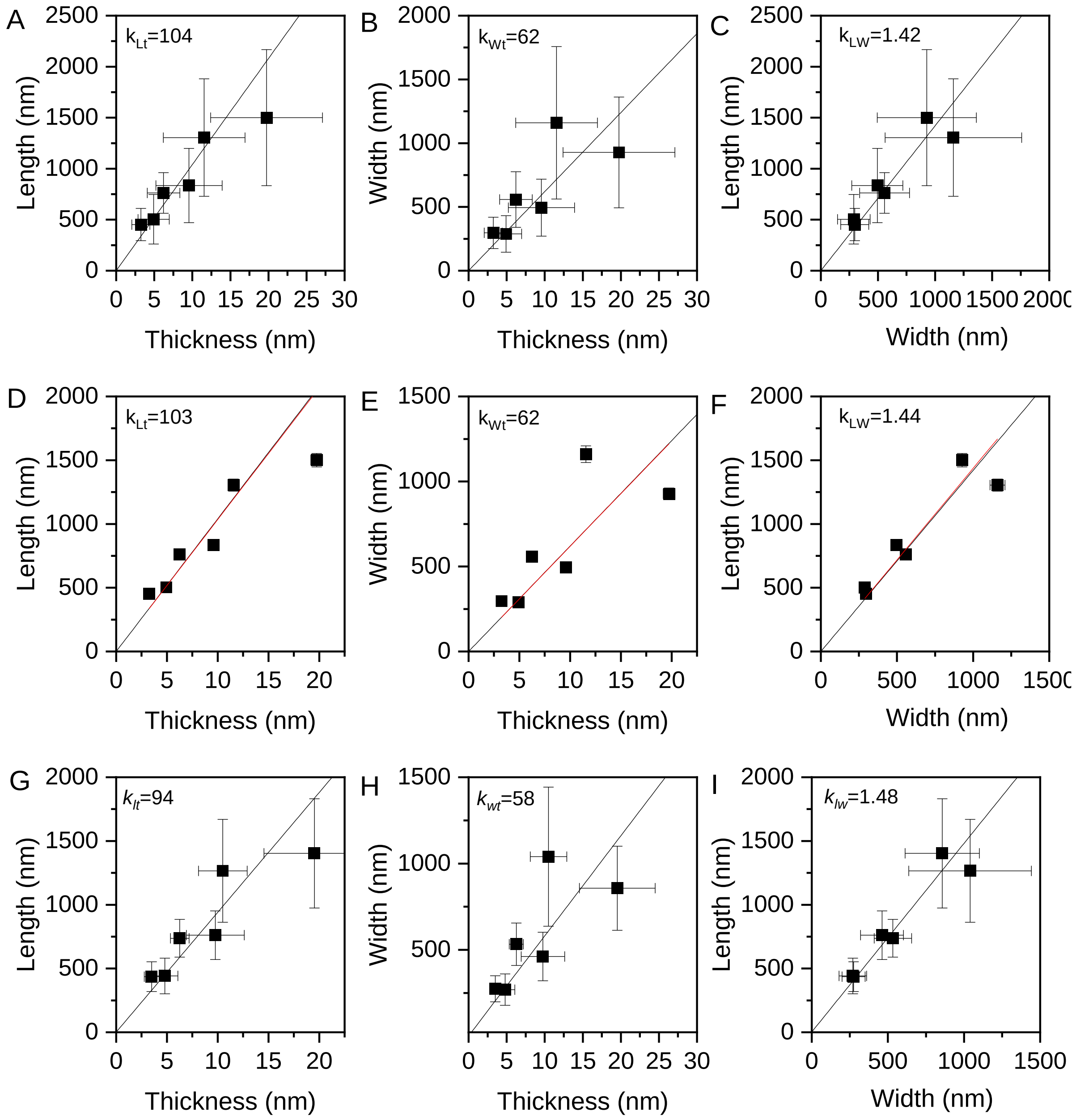

Figure S5: Inter-relationships between mean nanosheet length, width and thickness (corrected) as measured by AFM for six size-selected fractions of LPE graphene. The nanosheet aspect ratio, *k*, is given in each specific case as $k_{\mathrm{lt}} = l_{\mathrm{NS}} / t_{\mathrm{NS}}$, $k_{\mathrm{wt}} = w_{\mathrm{NS}} / t_{\mathrm{NS}}$ and $k_{\mathrm{lw}} = l_{\mathrm{NS}} / w_{\mathrm{NS}}$, where l_{NS}, w_{NS}, and t_{NS} are the nanosheet length, width and thickness, respectively. Aspect ratios are given in the panels. The data are presented in three different ways. In the first row **(A-C)** the data is presented as means ± the standard deviation (SD). This gives an impression of the width of the size distributions. In the second row **(D-F)** the data is presented as means ± the standard error. This gives an impression of the error in the mean. In the third row **(G-I)** the data is presented as median ± Median Absolute Deviation (MAD). This approach reduces the effect of skewed distributions which contain a longer than normal tail. It can be seen that the aspect

ratios do not vary significantly between mean and median analysis while the implied distribution widths don’t change much when using SD or MAD. In the main manuscript, we use the aspect ratios found in **(A-C)**.

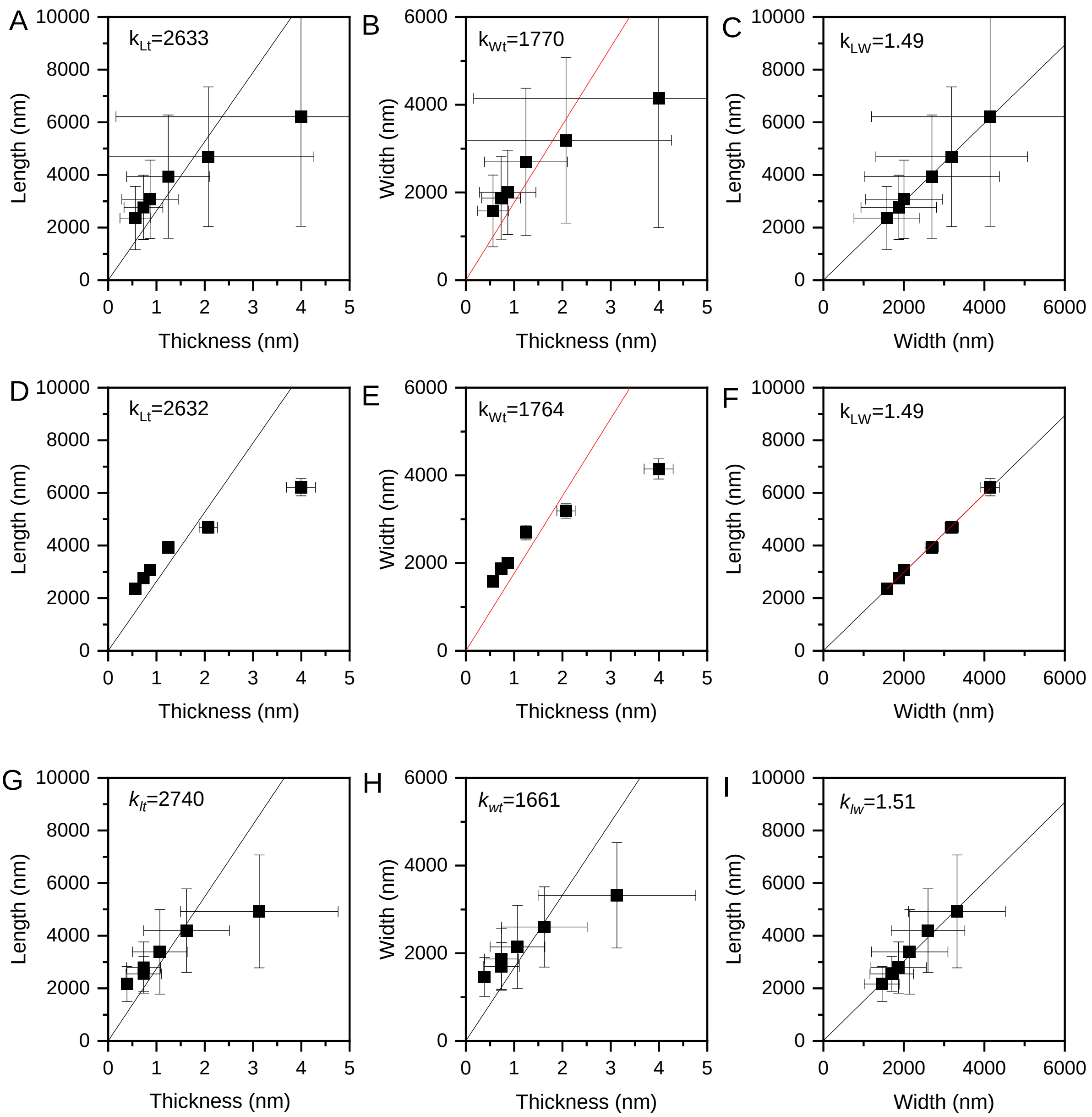

Figure S6: Inter-relationships between mean nanosheet length, width and thickness (corrected) as measured by AFM for six size-selected fractions of EE graphene. The nanosheet aspect ratio, *k*, is given in each specific case as $k_{\text{lt}} = l_{\text{NS}} / t_{\text{NS}}$, $k_{\text{wt}} = w_{\text{NS}} / t_{\text{NS}}$ and $k_{\text{lw}} = l_{\text{NS}} / w_{\text{NS}}$, where l_{NS}, w_{NS}, and t_{NS} are the nanosheet length, width and thickness, respectively. Aspect ratios are given in the panels. The data are presented in three different ways. In the first row **(A-C)** the data is presented as means ± the standard deviation (SD). This gives an impression of the width of the size distributions. In the second row **(D-F)** the data is presented as means ± the standard error. This gives an impression of the error in the mean. In the third row **(G-I)** the data is presented as median ± Median Absolute Deviation (MAD). This approach reduces the effect of skewed distributions which contain a longer than normal tail. It can be seen that the aspect

ratios do not vary significantly between mean and median analysis while the implied distribution widths are higher using SD versus MAD. We note that this data suggests the thickness of the largest fraction to be slightly overestimated. In the main manuscript, we use the aspect ratios found in **(A-C)**.

Supplementary Note 2: Surface Gradient Maps of LPE and EE Graphene Networks

To quantify the surface alignment of the LPE and EE graphene networks, the surfaces of each 3D volume generated by FIB-SEM nanotomography were first broken down into grids of equally sized square tiles for analysis. Tile sizes in the range of 50 – 1000 nm were considered for both networks. To determine the orientation of each surface tile, each one was approximated as a best-fit 2D plane using least squares fitting in MATLAB. This process is shown schematically for a surface tile in Figure. S7. This plane captures the average orientation of the network surface voxels within a specified tile with respect to the system coordinates, where the *Y*-axis represents the out-of-plane direction (perpendicular to the substrate). The polar angle between the normal vector describing each surface plane, $\hat{n}$, and the *Y*-axis is given by φ_{Surf} (Fig. S7). A surface tile where $\varphi_{\mathrm{Surf}} = 0°$ has a normal vector parallel to the out-of-plane *Y*-direction, meaning that portion of the surface is perfectly flat. Alternatively, tiles with values of $0° < \varphi_{\mathrm{Surf}} \leq 90°$ suggest local disorder on the network surface.

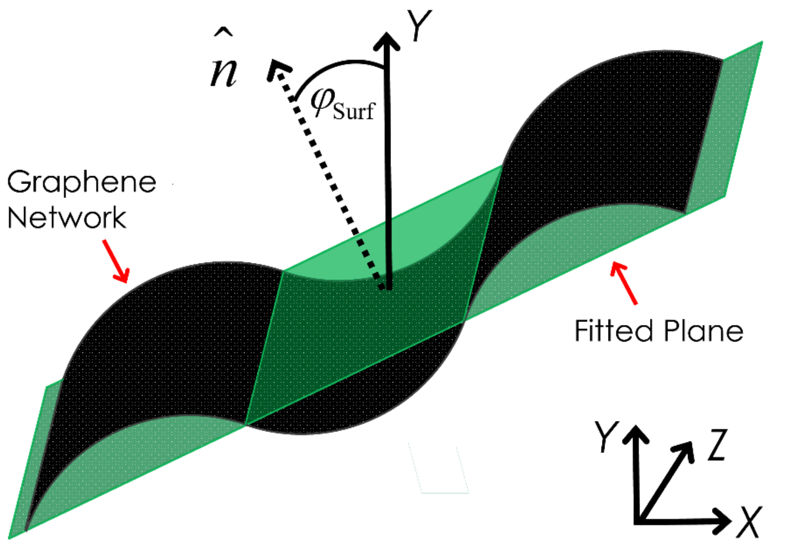

Figure S7: Measuring the orientation of the network surfaces. Schematic showing a best-fit plane applied to a network surface tile. The polar angle, φ_{Surf}, between the normal vector describing the plane, $\hat{n}$, and the *Y*-axis describes the average orientation of the network surface within the bounds of each tile.

By plotting heatmaps of φ_{Surf} as a function tile index (or position) on the network surface, the angular homogeneity and surface alignment can be visualised and quantitatively measured. This is shown for the printed EE and LPE graphene networks using a tile size of 330 × 330 nm in the main text (Fig. 3A-D). Heatmaps for a representative selection of tile sizes in the range of 100 – 1000 nm are shown for the EE and LPE graphene networks in Fig. S8A-E and Fig. S9A-E, respectively. To determine the influence of tile size on φ_{Surf}, we measured the mean squared surface angle, $\langle \varphi_{\mathrm{Surf}}^2 \rangle$, across all tiles in both the EE (Fig. S8F) and LPE (Fig. S9F) graphene networks. In both cases $\langle \varphi_{\mathrm{Surf}}^2 \rangle$ is seen to decay as the tile size is increased from 50

× 50 nm to 1000 × 1000 nm. This is to be expected, as averaging over larger tile sizes can artificially smooth the network surface when a plane is fitted to it, while smaller tile sizes can inflate $\langle \varphi_{\text{Surf}}^2 \rangle$. To ensure a fair comparison between both the EE and LPE networks, a tile size of 330 × 330 nm was chosen for surface analysis in the main text (Fig. 3A-D), which corresponds to half the measured LPE nanosheet length (i.e. $\text{Tile size} = 330\ \text{nm} = l_{NS,LPE}/2$).

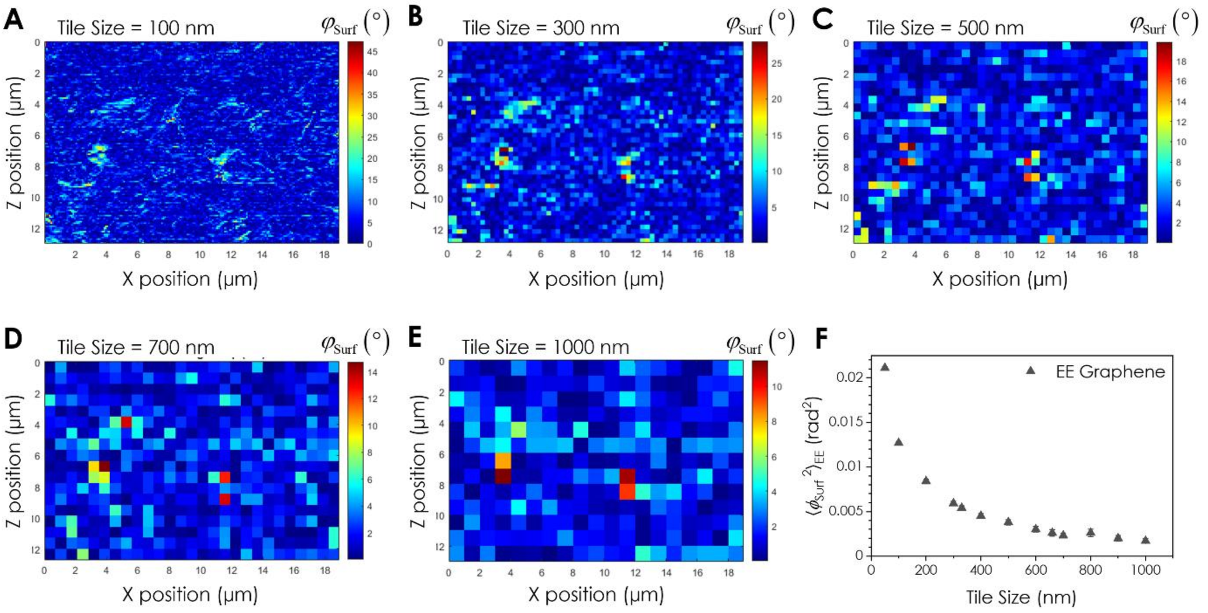

Figure S8: Surface gradient heatmaps for the EE graphene network. **(A-E)** Heatmaps of tile orientation as a function of position on the network surface for tile sizes of 100 nm **(A)**, 330 nm **(B)**, 500 nm **(C)**, 700 nm **(D)** and 1000 nm **(E)**. Each discrete square in **(A-E)** is a surface tile that has been fitted to extract the angle between its normal vector and the out-of-plane *Y*-direction, φ_{Surf}. **(F)** Plot of the mean squared surface angle, $\langle \varphi_{\text{Surf}}^2 \rangle_{\text{EE}}$, across all tiles in the EE graphene network as a function of tile size.

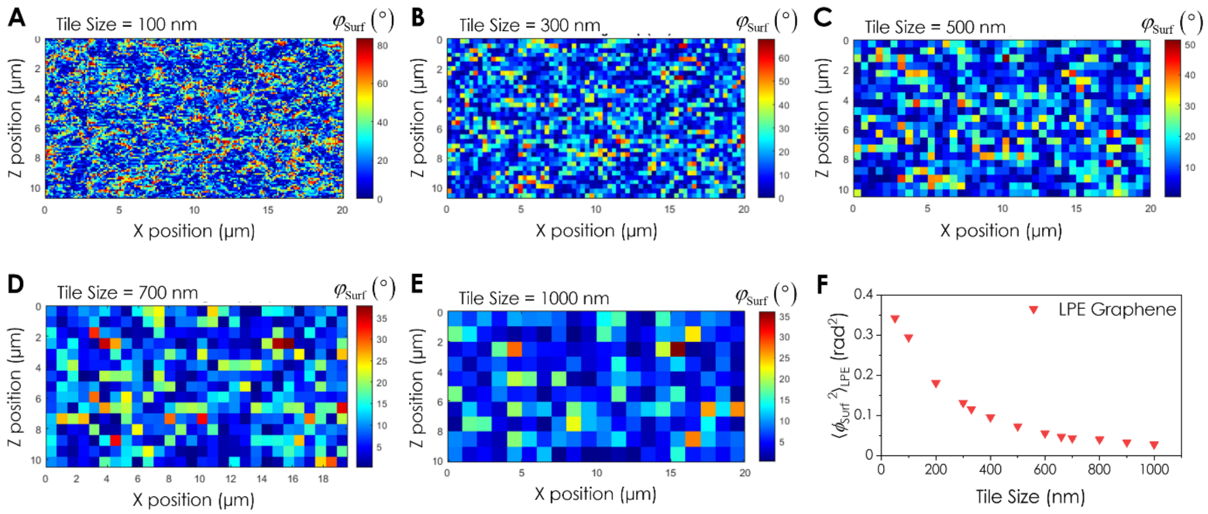

Figure S9: Surface gradient heatmaps for the LPE graphene network. **(A-E)** Heatmaps of tile orientation as a function of position on the network surface for tile sizes of 100 nm **(A)**, 330 nm **(B)**, 500 nm **(C)**, 700 nm **(D)** and 1000 nm **(E)**. Each discrete square in **(A-E)** is a surface tile that has been fitted to extract the angle between its normal vector and the out-of-plane *Y*-direction, φ_{Surf}. **(F)** Plot of the mean squared surface angle, $\langle \varphi_{Surf}^2 \rangle_{EE}$, across all tiles in the LPE graphene network as a function of tile size.

Supplementary Note 3: Nanosheet Orientation within the LPE Graphene Network

To determine the alignment of nanosheets within the LPE graphene network, as opposed to at the network surface, we performed analysis of the 3D volume using a previously reported method.[1] As the nanosheets in the LPE network are highly-connected (Fig. 2C, main text and Figure S10A), discrete 2D platelets were first separated by artificially introducing junctions between them using voxel spatial intensity relationships.[2] This was performed using a 3D Distance Transform Watershed operation within the MorphoLibJ plugin[3] in FIJI.[4] A chessboard distance transform was used as this equally weights in all directions and has shown improved performance for elongated particles, such as nanosheets.[5] The result of the 3D Distance Watershed Transform process is a network volume comprised of labelled nanosheet objects. An equivalent ellipsoid is then fitted to each labelled object based on its inertia tensor using the 3D Suite plugin in FIJI, which provides eigenvectors for each of the ellipsoid's primary axes.[6] A portion of the same LPE network, where the nanoplatelets have been replaced with equivalent ellipsoids for orientation analysis, is shown in Figure S10B (and Fig. 3E, main text).

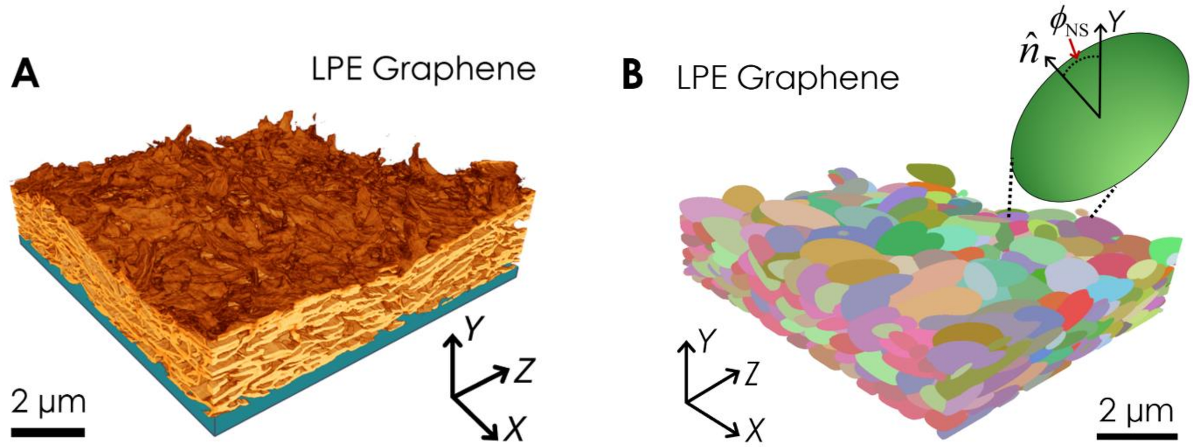

Figure S10: Nanosheet orientation from equivalent ellipsoids. **(A)** Portion of the reconstructed LPE graphene nanosheet volume. **(B)** A portion of the same LPE graphene network, where the aggregated nanoplatelets in the volume have been replaced with their equivalent ellipsoids. The eigenvector describing such an ellipsoid's minor axis is equivalent to the nanosheet normal vector, $\hat{n}$. Inset: Schematic showing that the angle between the nanosheet normal vector and the out-of-plane *Y*-direction is given by φ_{NS}.

As the equivalent ellipsoids shown in Figure S10B describe 2D nanosheets, or more generally restacked nanosheets with a common orientation, the normalised eigenvector describing smallest axis of each is in the same direction as the unit normal vector, $\hat{n}$, of the nanosheet. This is because $l_{\mathrm{NS}} \geq w_{\mathrm{NS}} \gg t_{\mathrm{NS}}$ for the LPE graphene in this study (Fig. S5). To find the angle between $\hat{n}$ and the *Y*-axis, we consider their dot product, where $\hat{n}$ and Y are unit vectors and φ_{NS} is the angle between them. As $\hat{n} = (n_X, n_Y, n_Z)$ and $Y = (0,1,0)$ it can be shown that $\hat{n} \cdot Y = n_{\mathrm{Y}} = \cos(\varphi_{\mathrm{NS}})$, meaning we can find the magnitude of the angle between a given nanosheet's normal vector and the *Y*-axis through $\varphi_{\mathrm{NS}} = \cos^{-1}(|n_{\mathrm{Y}}|)$. This angle can be

interpreted similarly to φ_{Surf} from the surface gradient analysis in the main text (Figure 3A-D); when $\varphi_{\text{NS}} = 0°$ the nanoplatelet within the network has a normal vector parallel to the out-of-plane *Y*-direction, meaning it is parallel to the substrate or horizontal. Alternatively, nanosheets/ellipsoids with values of $0° < \varphi_{\text{NS}} \leq 90°$ suggest local disorder within the network.

Supplementary Note 4: Statistical Robustness of Orientation Data from FIB-SEM NT Generated Volumes

While the lateral dimensions of the reconstructed 3D volumes appear small when compared to bulk scale techniques, we propose that the sample sizes of $N \sim 2000$ nanoplatelets for orientation analysis are representative of the global network. To quantitatively show this, we applied a bootstrap resampling approach to the orientation measurements extracted from our 3D images in the main text. Bootstrapping is a non-parametric statistical procedure that involves repeatedly extracting random samples (with replacement) from the original dataset to estimate the variability of statistics derived from it, without any assumptions about the dataset distribution[7]. For a finite dataset with N measurements, we randomly draw n values with replacement and measure the statistic of interest (i.e. mean, standard deviation, standard error, median and median absolute deviation). For each sample size of n values we repeat this step 100 times to build a distribution of that statistic for a sample size of n. From this distribution for a given n, both the mean and 95% confidence intervals of all 100 bootstrap replicates are calculated. This analysis is then repeated for a range of different sample sizes in the range $n \ll N$ to $n \sim N$. By plotting the mean and 95% confidence intervals as a function of sample size n, it is possible to see if these converge at a sample sizes below or approaching the experimental sample set N. If this is the case, the experimental sample size can be said to be representative and statistically robust.

We performed this analysis on the data for nanosheet orientation in the printed LPE graphene network shown in the main text (Fig. 3E-F, main text). Here, the nanosheet orientation distributions and the mean nanosheet orientation in the network were calculated by directly measuring 2538 nanosheets in the reconstructed volume (i.e. $N = 2538$). To test if this sample size was sufficient to be representative of the global network, we performed bootstrap analysis of the mean, median, standard deviation (Std. Dev.), standard error (SE), and median absolute deviation (MAD) in measured angles, φ_{NS}, for sample sizes ranging from $n = 10 – 2538$, with 100 bootstrap replicates for each sample size. As shown in Fig. S11A-D, the central values of the orientation statistics (mean, median, Std. Dev. and MAD) remain broadly constant across sample sizes from $n = 10 – 2538$ nanosheets. This is a clear indication that the sampling is unbiased, where even sampling tens of nanosheets gives mean, median, Std. Dev. and MAD estimates that are close to the values extracted for $N = 2538$ nanosheets. This suggests that the orientation distribution shown in the main text for printed LPE graphene (Fig. 3F) is spatially homogenous within the imaged volume. Furthermore, as the sample size is increased, the 95% confidence intervals are seen to narrow significantly (Fig. S11A-D), converging on their central values, which reflects improved precision in the distribution values. Crucially, this convergence occurs steadily, without considerable instability or drift. This is the statistical signature of a stable estimator and implies that the mean φ_{NS} values taken from $N = 2538$ nanosheets are statistically significant and reflect the true population values. Similar inferences can be made from the data for the standard error (SE) in the mean (Fig. S11E). Here, we see the central estimates for SE decay as sample size decreases, in line with the predicted $1/\sqrt{n}$ trend. This, along with the narrowing of the 95% confidence intervals with increasing n, highlights the improved precision and confidence in the extracted mean φ_{NS} value for the LPE graphene network (Fig. 3F, main text) for even a few hundred nanosheets.

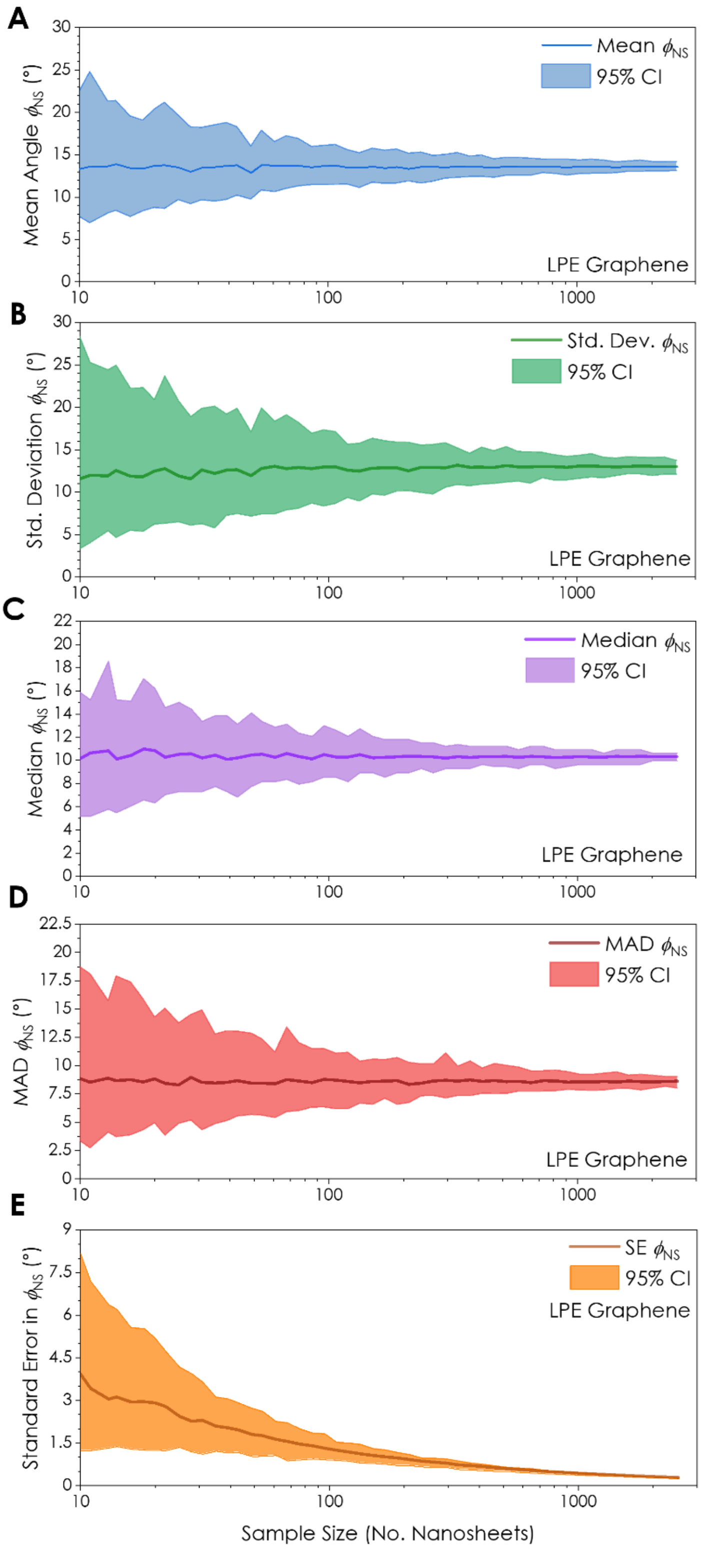

Figure S11: Bootstrap analysis of orientation statistics for spray-coated LPE graphene. Plots of the mean **(A)**, standard deviation **(B)**, median **(C)**, median absolute deviation (MAD) **(D)**, and standard error in the mean **(E)** in nanosheet orientation, φ_{NS}, as a function of sample size, n. The data in **(A-E)** is calculated for sample sizes that range from $n = 10 - 2538$ using the nanosheet orientation results presented in Fig. 3F (main text). The central estimates in the mean, standard deviation, median and MAD **(A-D)** are seen to remain constant across all sample sizes, while the 95% confidence intervals narrow significantly with increasing sample size. The predicted $1/\sqrt{n}$ scaling in standard error with increasing sample size is shown in **(E)**.

Such behaviour is consistent with what might be expected for a printed nanosheet network, where there is global alignment with some local disorder[8] – barring any printing defects. Care was taken to image representative regions of the printed films to alleviate this. This structural homogeneity is also supported by previous work within our group, where printed networks of LPE nanosheets for different materials were shown to have spatially invariant morphological properties, such as tortuosity factor, in different in-plane directions[1]. Taken together, the data in Fig. S11A-E clearly suggests that the sample size of N = 2538 nanosheets is more than sufficient to yield reliable and representative estimates of the nanosheet orientation within the network shown in the main text (Fig. 3E-F, main text).

In summary, while the FIB-SEM NT technique is localised, it offers statistically robust and high resolution nanosheet orientation data at the nanoscale that is inaccessible to other macroscopic techniques. Crucially, this allows morphological parameters, including nanosheet alignment, to be measured at length scales that are relevant to inter-nanosheet junctions and charge transport through the network. Nonetheless, we agree that future work including macroscopic techniques such as XRD or polarised Raman spectroscopy could help benchmark local and global orientation metrics across different length scales to further reinforce the statistically robust values provided by FIB-SEM NT.

Supplementary Note 5: Inter-nanosheet Junctions in Printed LPE Graphene Networks

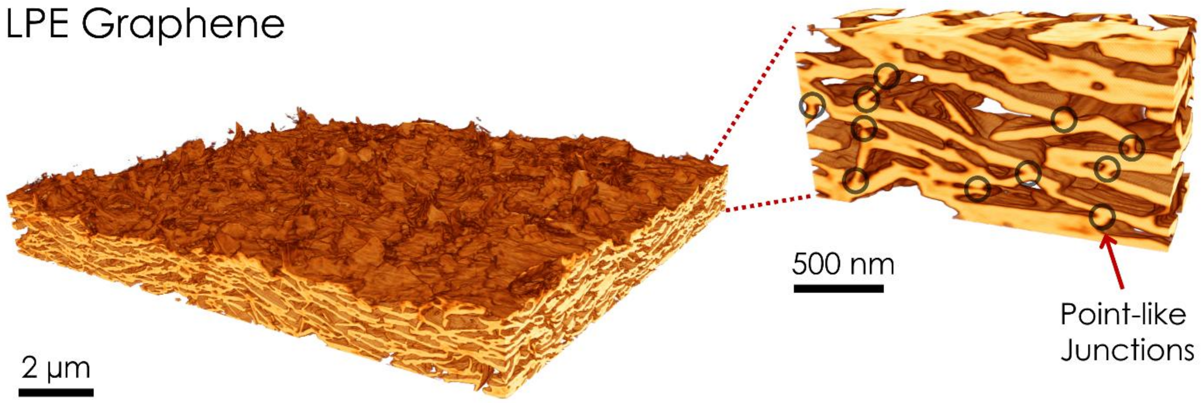

Figure S12: Point-like junctions in printed LPE graphene networks. Portion of the reconstructed 3D volume for the LPE graphene network shown in the main text (Figure 3C). A magnified region showing that the rigid, low-aspect ratio LPE nanosheets generally form point-like junctions, where nanosheet vertices are in contact with the basal planes of other nanosheets.

Supplementary References

1 Gabbett, C. *et al.* Quantitative analysis of printed nanostructured networks using high-resolution 3D FIB-SEM nanotomography. *Nature Communications* **15**, doi:10.1038/s41467-023-44450-1 (2024).

2 Ghoshal, D. & PratimAcharjya, P. Watershed Segmentation based on Distance Transform and Edge Detection Techniques. *International Journal of Computer Applications* **52**, 6-10, doi:10.5120/8259-1792 (2012).

3 Legland, D., Arganda-Carreras, I. & Andrey, P. MorphoLibJ: integrated library and plugins for mathematical morphology with ImageJ. *Bioinformatics*, doi:10.1093/bioinformatics/btw413 (2016).

4 Schindelin, J. *et al.* Fiji: an open-source platform for biological-image analysis. *Nature Methods* **9**, 676-682, doi:10.1038/nmeth.2019 (2012).

5 Chen, Q., Yang, X. & Petriu, E. M. in *Proceedings of the 3rd IEEE international workshop on haptic, audio and visual environments and their applications* Vol. 2 111-116 (2004).

6 Ollion, J., Cochennec, J., Loll, F., Escudé, C. & Boudier, T. TANGO: a generic tool for high-throughput 3D image analysis for studying nuclear organization. *Bioinformatics* **29**, 1840-1841, doi:10.1093/bioinformatics/btt276 (2013).

7 Efron, B. & Tibshirani, R. J. *An Introduction to the Bootstrap*. (1994).

8 Piatti, E. *et al.* Charge transport mechanisms in inkjet-printed thin-film transistors based on two-dimensional materials. *Nature Electronics* **4**, 893-905, doi:10.1038/s41928-021-00684-9 (2021).
